# Unique Information Through the Lens of Channel Ordering: An Introduction and Review

**DOI:** 10.3390/e27010029

**Published:** 2025-01-01

**Authors:** Pradeep Kr. Banerjee

**Affiliations:** Institute for Data Science Foundations, Blohmstraße 15, 21079 Hamburg, Germany; pradeep.banerjee@tuhh.de

**Keywords:** comparison of channels, unique information, Blackwell order, information-theoretic cryptography, secret key rate, secrecy monotones, synergy, redundancy, Le Cam deficiency, resource theories

## Abstract

The problem of constructing information measures with a well-defined interpretation is of fundamental significance in information theory. A good definition of an information measure entails certain desirable properties while also providing answers to operational problems. In this work, we investigate the properties of the unique information, an information measure that quantifies a deviation from the Blackwell order. Beyond providing an accessible introduction to the topic from a channel ordering perspective, we present a novel resource-theoretic characterization of unique information in a cryptographic task related to secret key agreement. Our operational view of unique information entails rich physical intuition that leads to new insights into secret key agreement in the context of non-negative decompositions of the mutual information into redundant and synergistic contributions. Through this lens, we illuminate new directions for research in partial information decompositions and information-theoretic cryptography.

## 1. Introduction

Shannon’s pioneering work [1] characterized the capacity of a physical channel by way of maximum mutual information. Since then, information theory has had a special relation to communication engineering, even though ideas and tools from information theory have been successfully applied in many other research fields, such as cryptography, statistics, machine learning, complex systems, and biology, to name a few.

Despite significant progress in information theory, many fundamental questions remain regarding the nature of information. One of the primary challenges is that information is not a conserved quantity, making it difficult to track and describe its distribution across composite systems. A composite system consists of multiple interacting subsystems, each of which may hold *unique* (or exclusive) information, or share *redundant* (or shared) information. Additionally, there are cases where some information is not directly accessible to any individual subsystem but can only be determined by considering the entire system. For example, a checksum for a set of digits can only be computed when all the digits are known. Such *synergistic* effects are especially relevant in cryptography, where the objective is for the encrypted message to reveal no information about the original message without the corresponding key.

How should the amount of unique, shared, and synergistic information be measured? This question can be approached from two different points of view, namely, the *axiomatic* and the *operational* [2]. In an *axiomatic* approach, one posits certain desirable properties that a measure of information should satisfy. This point of view goes back to Shannon [1], who showed that his definition of entropy is the only one that satisfies certain intuitively appealing properties. Shannon notes that such an axiomatic characterization is “*in no way necessary for the theory*” but “*lends a certain plausibility*” to the definitions and that the “*real justification of these definitions, however, will reside in their implications*” [1]. Thus, in Shannon’s view, the ultimate criterion for accepting some quantity as a measure of information is whether it provides answers to interesting problems. This is an *operational* or *pragmatic* view of information. For example, Shannon’s coding theorems endow the entropy and mutual information with concrete meaning in operational tasks related to data compression and transmission. Rényi [2,3] and Csiszár [4,5] comment that for problems that lay outside the scope of these theorems, both the axiomatic and the operational points of view deserve attention and can, in fact, be used to “control” or inform the other when constructing new measures of information. Understanding the properties of these measures helps clarify the fundamental limits of operational problems. Dually, analyzing such problems motivates the quest for new information measures.

This review investigates the properties of the unique information (UI), an information measure introduced by Bertschinger et al. [6], which quantifies deviations from the Blackwell order. We adopt Shannon’s pragmatic stance, focusing on an operational view of unique information from a channel ordering perspective. This builds on the original definition of UI in [6], which is motivated by the idea that unique information should be “useful”. Bertschinger et al. formalized this idea in terms of decision problems: Whenever Bob has unique information about something Alice knows (which is not accessible to Eve), there is a decision problem in which Bob has an advantage over Eve. By leveraging tools from resource theories [7,8,9,10,11], we provide a concrete formalization of this conceptual framework.

Resource theories provide an abstract operational framework for studying what physical transformations between a given set of objects are possible under restrictions that follow from the nature of the system under investigation. Within this framework, resources are measured by *monotones*, quantities that do not increase under allowed operations. We present a novel resource-theoretic characterization of UI in a fundamental cryptographic task related to secret key agreement, showing that UI functions similarly to classical *secret key rates* [12,13,14,15], and is in fact a monotone that quantifies the “resourcefulness” or secrecy content of a source distribution. This operational characterization not only extends the applicability of UI in cryptographic settings but also opens up new avenues for its study within resource theory.

Our resource-theoretic approach represents a significant departure from existing frameworks on bivariate partial information decompositions, offering a fresh perspective on how UI can be leveraged in the broader context of information-theoretic cryptography. While most existing approaches have focused on the shared information within these decompositions using an axiomatic framework, we shift the focus to an operational view of UI, grounded in the context of channel preorders. Shannon emphasized that the value of an information measure should be judged by its implications in practical tasks, rather than its adherence to abstract properties alone. Following this viewpoint, we argue that UI’s significance emerges most clearly when applied in concrete settings like decision-making or cryptographic problems, where operational utility takes precedence over purely axiomatic considerations.

This work serves as both a review and a formalization of existing research on UI and related measures based on channel orderings, with a particular focus on interpreting these insights through the lens of resource theories. The focus of this review is primarily on measures akin to UI, particularly those rooted in channel orderings, and does not extend to other types of information measures that fall outside this framework. We draw extensively from previously published works [16,17,18,19,20,21] and integrate key insights from unpublished portions of the author’s PhD thesis [22], which are not available in the public domain. By synthesizing these contributions, this review not only provides a comprehensive overview of prior research on UI and channel preorders, but also introduces novel resource-theoretic perspectives, offering a fresh and compelling advancement in the study of bivariate partial information decompositions and information-theoretic cryptography.

**Outline.** The paper is organized as follows: Section 2 provides a brief review of prior work on non-negative bivariate information decompositions and presents a formal description of the problem, with a focus on the properties of the function UI as introduced by Bertschinger et al. [6]. Section 3 offers a self-contained exposition on channel orderings in information theory. In Section 4, we review Le Cam deficiencies and their generalizations [23,24,25], which exhibit properties analogous to the UI. These deficiencies quantify the cost of approximating one channel by another through randomization, capturing deviations from output- and input-degraded channel orderings. This provides insight into the distinctions between the bivariate decompositions of Bertschinger et al. [6] and Harder et al. [26]. In Section 5, we review the operational significance of UI in a cryptographic task related to secret key agreement [16,17]. Finally, Section 6 presents a novel resource-theoretic characterization of the main results from Section 5, demonstrating that the UI serves as a resource “monotone” quantifying the secrecy content of a given distribution under a specific class of allowed operations.

## 2. Bivariate Partial Information Decompositions

**Notation and conventions.** We shall use notation that is commonly used in information theory [27,28]. We assume that random variables *S*, *Y*, *Z*, etc., are finite, as are all other random variables in this work. The set of all probability measures on a finite set S is denoted by $P_{S}$. A *channel* μ from S to Z is a family $\mu ={\left\{\mu_{s}\right\}}_{s\in S}$ of probability distributions on Z, one for each possible input s∈S. We write M(S;Z) to denote the space of all channels from S to Z. Given two channels, μ∈M(S;Z) and ρ∈M(Z;Y), the composition ρ∘μ∈M(S;Y) of μ with ρ is defined as follows: $\rho \circ \mu_{s}\left(y\right)=\sum_{z\in Z}\rho_{z}\left(y\right)\mu_{s}\left(z\right)$ for all s∈S, z∈Z. A *binary symmetric channel* with parameter *p*, denoted as BSC(p), is a channel from S={0,1} to Y={0,1} that flips each bit independently with some error probability $p\in [0,\frac{1}{2}]$. A *binary erasure channel* on S={0,1} with erasure probability ϵ∈[0,1], denoted as BEC(ϵ), is a channel from S to Y=S∪{e} such that Y=S with probability 1−ϵ and Y=e with probability ϵ. Given two distributions *P* and *Q*, the Kullback–Leibler (KL) divergence from *P* to *Q* is denoted as D(P∥Q). H(S) denotes the Shannon entropy of random variable *S*. h(·) is the binary entropy function, h(p)=−plogp−(1−p)log(1−p) for p∈(0,1) and h(0)=h(1)=0.

The mutual information of two random variables *S* and *Y* is defined as

(1) $$\begin{array}{c} I(S;Y)=H(S)+H(Y)-H(SY). \end{array}$$

*I* measures the total amount of correlation between *S* and *Y* and possesses the following key properties [28,29]:

(2a) $$\begin{array}{cccc} \; & I(S;Y)=I(Y;S) & \; & \;(\mathrm{symmetry}), \end{array}$$

(2b) $$\begin{array}{cccc} \; & I(S;Y)\geq 0;\;I(S;Y)=0\Leftrightarrow S\mathrm{is}\mathrm{independent}\mathrm{of}\;Y & \; & \;(\mathrm{non}\text{-}\mathrm{negativity}), \end{array}$$

(2c) $$\begin{array}{cccc} \; & I(S;YZ)\geq I(S;Y) & \; & \;(\mathrm{strong}\mathrm{subadditivity}). \end{array}$$

Strong subadditivity is equivalent to the non-negativity of the conditional mutual information:

$$\begin{array}{c} I(S;Z|Y)\geq 0;\;I(S;Z|Y)=0\Leftrightarrow S-Y-Z, \end{array}$$

where S−Y−Z denotes that *S*, *Y*, and *Z* form a Markov chain in that order. Strong subadditivity also implies the following key property of the mutual information, namely, its monotonicity with respect to data processing

$$\begin{array}{c} S-Z-Y\Rightarrow I(S;Z)\geq I(S;Y),\;with\;equality\;if\;and\;only\;if\;S-Y-Z\\ (data\;processing\;inequality). \end{array}$$

Another integral property of the mutual information is the following equality, which is called the chain rule:

(3) $$\begin{array}{ccc} I(S;YZ)=I(S;Y)+I(S;Z|Y) & \; & \;(\mathrm{chain}\mathrm{rule}). \end{array}$$

In general, conditioning on an additional random variable can either increase or decrease the mutual information. We consider three canonical distributions to illustrate this point. Each of these distributions capture a fundamentally different kind of interaction between three jointly distributed random variables.

**Example** **1.** *The* Rdn *,* Xor *, and the* Copy *distributions.*

Rdn*: If S, Y, and Z are uniformly distributed binary random variables with S=Y=Z, then conditioning on Z decreases the mutual information between S and Y. This is an instance of a purely* redundant *interaction where Y and Z convey the same information about S.*
Xor*: If S and Y are independent binary random variables, and Z=S⊕Y (where *⊕* denotes the binary *Xor* operation), then conditioning on Z increases the mutual information between S and Y. This is an instance of a purely* synergistic *interaction where neither Y nor Z individually conveys any information about S, but jointly, they fully determine S.*
Copy*: If Y and Z are independent uniformly distributed binary random variables, and S=(Y,Z) then I(S;Y)=I(S;Y|Z)=H(Y)=1 bit, and I(S;Z)=I(S;Z|Y)=H(Z)=1 bit. This is an instance of an interaction that is neither redundant nor synergistic, but purely* unique*, for now, Y and Z each uniquely conveys 1 bit of information about S.*

In general, all three forms of interaction—unique, redundant, and synergistic—can coexist simultaneously. Our goal is to disentangle the individual contributions to the mutual information between *S* and (Y,Z) arising from these interactions. Specifically, we distinguish *S* as the *target* variable of interest, with *Y* and *Z* serving as *predictor* variables.

Let $\tilde{UI}$, $\tilde{SI}$, and $\tilde{CI}$ be non-negative functions that depend continuously on the joint distribution of (S,Y,Z). The mutual information between *S* and *Y* can be decomposed into two components: information *Y* has about *S* that is *unknown* to *Z* (referred to as the *unique* or *exclusive* information of *Y* with respect to *Z*), and information *Y* has about *S* that is *known* to *Z* (referred to as the *shared* or *redundant* information). This decomposition is given by the following:

(4) $$\begin{array}{c} I\left(S;Y\right)=\underset{\mathrm{unique}\;Y\;\mathrm{with}\;\mathrm{respect}\;\mathrm{to}\;Z}{\underset{︸}{\tilde{UI}\left(S;Y\backslash Z\right)}}+\underset{\mathrm{shared}\;\left(\mathrm{redundant}\right)}{\underset{︸}{\tilde{SI}\left(S;Y,Z\right)}}. \end{array}$$

Conditioning on *Z* eliminates the shared information but introduces *complementary* (or *synergistic*) information arising from the interaction between *Y* and *Z*. This is expressed as follows:

(5) $$\begin{array}{c} I\left(S;Y|Z\right)=\underset{\mathrm{unique}\;Y\;\mathrm{with}\;\mathrm{respect}\;\mathrm{to}\;Z}{\underset{︸}{\tilde{UI}\left(S;Y\backslash Z\right)}}+\underset{\mathrm{complementary}\;\left(\mathrm{synergistic}\right)}{\underset{︸}{\tilde{CI}\left(S;Y,Z\right)}}. \end{array}$$

**Figure 1:**
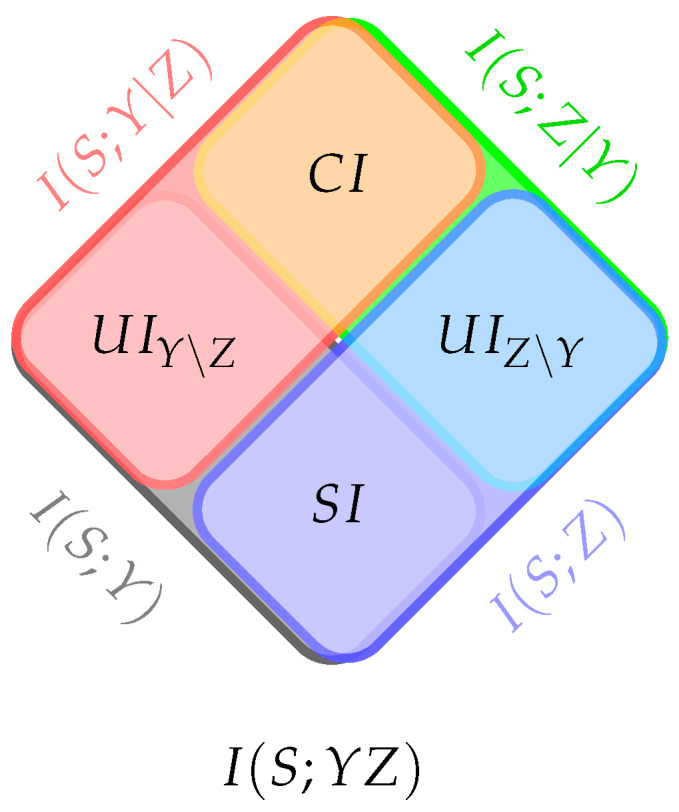
An illustration of the information decomposition in Equations (4)–(6).

The unique information can be interpreted either as the conditional mutual information without synergy or as the mutual information without redundancy. Applying the chain rule for mutual information, the total mutual information between *S* and (Y,Z) can be decomposed into four distinct terms, as illustrated in Figure 1:

(6) $$\begin{array}{c} I\left(S;YZ\right)=\tilde{UI}\left(S;Y\backslash Z\right)+\tilde{SI}\left(S;Y,Z\right)+\tilde{UI}\left(S;Z\backslash Y\right)+\tilde{CI}\left(S;Y,Z\right). \end{array}$$

Equations (4)–(6) leave only a single degree of freedom; i.e., it suffices to specify either a measure for $\tilde{SI}$, for $\tilde{CI}$, or for $\tilde{UI}$. Any definition of the measure $\tilde{UI}$ fixes two of the terms in (6), which, in turn, also determines the other terms by (4) and (5). This gives rise to the following *consistency condition*:

(7) $$\begin{array}{c} I\left(S;Y\right)+\tilde{UI}\left(S;Z\backslash Y\right)=I\left(S;Z\right)+\tilde{UI}\left(S;Y\backslash Z\right). \end{array}$$

The *coinformation* [30] is defined as the difference between the shared and synergistic information. It serves as a symmetric measure of correlation among three random variables:

(8) $$\begin{array}{c} CoI\left(S;Y;Z\right)=\tilde{SI}\left(S;Y,Z\right)-\tilde{CI}\left(S;Y,Z\right)=I\left(S;Y\right)-I\left(S;Y|Z\right). \end{array}$$

Coinformation is called *interaction information* (with a change of sign) in [31] and *multiple mutual information* in [32]. The Xor distribution in Example 1 shows that CoI can be negative. Coinformations and entropies are related by a Möbius inversion [30]. Equation (8) can equivalently be written as a linear combinations of entropies:

(9) $$\begin{array}{c} CoI(S;Y;Z)=H(S)+H(Y)+H(Z)-H(SY)-H(SZ)-H(YZ)+H(SYZ). \end{array}$$

Yeung [33] discusses properties of the CoI as a signed measure using analogies between sets and random variables. Te Sun [34] studies the more general question of what linear combinations of entropies are always non-negative.

Yet, another way to express the CoI is in terms of mutual informations:

(10) $$\begin{array}{c} CoI(S;Y;Z)=I(S;Y)+I(S;Z)-I(S;YZ). \end{array}$$

Equation (10) shows that CoI can be interpreted as a measure of the “extensivity” of mutual information, i.e., how the mutual information increases as we combine *Y* and *Z* [35]: If CoI=0, then the mutual information is exactly extensive in the sense that I(S;YZ) is the sum of the mutual informations I(S;Y) and I(S;Z). If CoI>0, then the mutual information is subextensive and the shared component dominates the synergistic component. Conversely, if CoI<0, then the mutual information is superextensive and the synergistic component dominates the shared component.

*Coinformation* is a widely utilized measure in neuroscience and related fields, with positive values interpreted as redundancy and negative values as synergy [36,37,38,39,40,41,42,43,44,45]. However, it cannot detect interactions where redundancy and synergy are perfectly balanced [46].

The *correlational importance*, a non-negative measure for evaluating the role of correlations in neural coding [47,48,49] (see also [50]), aligns conceptually with complementary information. Notably, it can sometimes exceed the total mutual information, as demonstrated in specific examples [51].

Non-negative decompositions of the form (4)–(6) that seek to disentangle the synergistic and redundant contributions to the total information that a pair of predictors convey about the target *S* were first considered by Williams and Beer [46]. Some notable follow-up works include [6,26,52,53,54,55,56,57,58,59]. For the general case of *k* finite predictor variables, Williams and Beer proposed the *partial information lattice* framework to decompose the mutual information between the target and predictors into a sum of non-negative terms corresponding to the different ways in which combinations of the predictor variables convey shared, unique, or complementary information about *S*. The lattice is a consequence of certain natural properties of the shared information, sometimes called the *Williams–Beer axioms*. The underlying idea is that any information about *S* can be classified according to “who knows what”, i.e., which information about *S* is shared by which subsets of the predictors [59]. Specializing to the bivariate case (k=2), the Williams–Beer axioms only put crude bounds on the values of the functions $\tilde{SI}$, $\tilde{UI}$, and $\tilde{CI}$ in (4)–(6). Additional axioms have been proposed in [26,60]. See Appendix A for a brief review of these axioms. Unfortunately, some of these axioms contradict each other, and the question for the right axiomatic characterization of shared information is still open.

Bertschinger et al. [6] proposed a pragmatic approach to decompositions of the form (4)–(6) based on the idea that if *Y* has unique information about *S* with respect to *Z*, then there must be a situation or task where such unique information is useful. This idea is formalized in terms of decision problems. We recall the definitions in [6].

**Definition** **1** ([6])**.** *For some finite state spaces Y,Z, and S, let $P_{S\times Y\times Z}$ be the set of all joint distributions of (S,Y,Z). Given $P\in P_{S\times Y\times Z}$, let*

(11) $$\begin{array}{c} \Delta_{P}:\;=\left\{Q\in P_{S\times Y\times Z}:Q_{SY}\left(s,y\right)=P_{SY}\left(s,y\right)\;and\;Q_{SZ}\left(s,z\right)=P_{SZ}\left(s,z\right)\right\} \end{array}$$

*denote the set of all joint distributions of (S,Y,Z) that have the same marginals on (S,Y) and (S,Z) as P. The unique information that Y conveys about S with respect to Z is defined as*

(12a) $$\begin{array}{cc} \; & UI\left(S;Y\backslash Z\right)=\min_{Q\in \Delta_{P}}I_{Q}\left(S;Y|Z\right), \end{array}$$

*where the subscript Q in $I_{Q}$ denotes the joint distribution on which the function is computed. Specifying* (12a) *fixes the other three functions in* (6)*, which are then*

(12b) $$\begin{array}{cc} \; & UI\left(S;Z\backslash Y\right)=\min_{Q\in \Delta_{P}}I_{Q}\left(S;Z|Y\right), \end{array}$$

(12c) $$\begin{array}{cc} \; & SI\left(S;Y,Z\right)=\max_{Q\in \Delta_{P}}CoI_{Q}\left(S;Y;Z\right), \end{array}$$

(12d) $$\begin{array}{cc} \; & CI(S;Y,Z)=I(S;Y|Z)-UI(S;Y\backslash Z). \end{array}$$

The functions UI, SI, and CI are non-negative and satisfy (4)–(6) (and hence (7)). Furthermore, the function SI satisfies the bivariate Williams–Beer axioms [6] (see Appendix A):

(13) $$\begin{array}{cccc} \; & SI(S;Y,Z)=SI(S;Z,Y) & \; & \;(\mathrm{symmetry}),\\ \; & SI(S;Y)=I(S;Y) & \; & \;(\mathrm{self}\text{-}\mathrm{redundancy}),\\ \; & SI(S;Y,Z)\leq SI(S;Y)\mathrm{with}\mathrm{equality}\mathrm{if}\;Z\mathrm{is}a\mathrm{function}\mathrm{of}\;Y & \; & \;(\mathrm{bivariate}\mathrm{monotonicity}). \end{array}$$

The definition of the function UI is rooted in a notion of channel domination due to Blackwell [61]. Intuitively, one channel dominates another if the latter can be “simulated” by the former by some stochastic degradation. UI satisfies the following key property which we call the *Blackwell property* (see Definition 10):

**Lemma** **1** (Vanishing UI [6], Lemma 6)**.** *For a given joint distribution $P_{SYZ}$, UI(S;Y\Z) vanishes if and only if there exists a random variable $Y^{\prime }$ such that $S-Z-Y^{\prime }$ is a Markov chain and $P_{SY^{\prime }}=P_{SY}$.*

Blackwell’s theorem [61,62] establishes that UI(S;Y\Z)=0 is equivalent to the assertion that, for any decision problem involving the prediction of *S*, having access to *Z* provides the same predictive capability as having access to *Y* (see Theorem 1).

Given (S,Y,Z)∼P, let

(14) $$\begin{array}{c} Q^{0}\left(s,y,z\right)=\left\{\begin{array}{cc} \frac{P(s,y)P(s,z)}{P(s)}, & \;\mathrm{if}\;P(s)>0,\\ 0, & \;\mathrm{else}. \end{array}\right. \end{array}$$

Observe that $Q^{0}\in \Delta_{P}$. Moreover, $Q^{0}$ defines a Markov chain Y−S−Z. The following lemma gives conditions under which the function SI vanishes:

**Lemma** **2** (Vanishing SI [6], Lemma 9)**.** *SI vanishes if and only if $I_{Q^{0}}\left(Y;Z\right)=0$.*

Lemma 3 characterizes the quantities UI, SI, and CI among alternative definitions of information decompositions.

**Lemma** **3** ([6], Lemma 3)**.** *Let $\tilde{UI}\left(S;Y\backslash Z\right)$, $\tilde{UI}\left(S;Z\backslash Y\right)$, $\tilde{SI}\left(S;Y,Z\right)$, and $\tilde{CI}\left(S;Y,Z\right)$ be non-negative functions on $P_{S\times Y\times Z}$ satisfying equations *(4)–(6)*, and assume that the following holds:*

(∗) *$\tilde{UI}$ depends only on the marginal distributions of the pairs (S,Y) and (S,Z).*

*Then, $\tilde{UI}\leq UI$, $\tilde{SI}\geq SI$, and $\tilde{CI}\geq CI$ with equality if and only if there exists $Q\in \Delta_{P}$ such that ${\tilde{CI}}_{Q}\left(S;Y,Z\right)=0$.*

By Lemma 3, (12a–12d) is the *only* information decomposition that satisfies (∗) and the following property:

(∗∗) For each P∈Δ, there is $Q\in \Delta_{P}$ with $CI_{Q}\left(S;Y,Z\right)=0$.

Assumption (∗) in Lemma 3 is motivated by the Blackwell property, which also depends only on the marginal distributions of the pairs (S,Y) and (S,Z).

Given (S,Y,Z)∼P, let

(15) $$\begin{array}{c} Q^{*}\in \underset{Q\in \Delta_{P}}{arg min}I_{Q}\left(S;Y|Z\right). \end{array}$$

By definition, $I_{Q^{*}}\left(S;Y|Z\right)=UI\left(S;Y\backslash Z\right)$. The distribution $Q^{*}$ is called a *minimum synergy distribution* as

(16) $$\begin{array}{c} CI_{P}\left(S;Y,Z\right)=0\mathrm{if}\mathrm{and}\mathrm{only}\mathrm{if}\;P\in \underset{Q\in \Delta_{P}}{arg min}I_{Q}\left(S;Y|Z\right). \end{array}$$

Although theoretically promising, the operational significance of the UI is not immediately evident, except in cases where it vanishes, reflecting the Blackwell property. One of our main aims is to address this gap by examining UI’s operational relevance in practical model systems through the following key observations:

- The Blackwell relation induces a partial order on channels with the same input alphabet. Most channels are incomparable, meaning one cannot always simulate another by degradation. In such cases, UI quantifies the degree of deviation from simulating one channel by another.
- Weaker notions of channel comparison, such as the “less noisy” property [63], have operational significance through vanishing $C_{S}$, where $C_{S}$ is the secrecy capacity of the wiretap channel [64,65]. Similar to how $C_{S}$ measures deviation from the less noisy order, a nonvanishing UI quantifies a deviation from the Blackwell order and bounds operational quantities in secret key agreement tasks. In particular, UI acts as a *secrecy monotone*, never increasing under local operations in one-way secret key agreement protocols, making it an upper bound on the *one-way secret key rate* $S_{\to }$ [66]. This endows the UI with operational significance.
- Finally, the best-known upper bounds on the *two-way secret key rate* $S_{↔}$ involve a secret key decomposition [67]. We show that UI satisfies a similar property, ensuring UI is never greater than the best-known computable upper bound on $S_{↔}$. We conjecture that UI serves as a lower bound on $S_{↔}$ and identify a class of distributions where they coincide.

## 3. Comparison of Channels

Given two channels that convey information about the same random variable, a natural question is “which channel is better?”. Depending on the task at hand, some orderings are more natural or mathematically more appealing than others. For example, ordering channels according to their capacity is often too coarse to be useful in practice. In a seminal paper [61], David Blackwell introduced an ordering of channels in terms of risks of statistical decision rules. Blackwell showed that such an ordering can be equivalently characterized in terms of a purely probabilistic relation between the channels. Blackwell formulated his result in terms of a decision problem, where a decision maker or agent reacts to the outcome of a statistical experiment. In information-theoretic parlance, a statistical experiment is just a noisy channel [4,25]. Shannon [68] independently introduced a criterion for ordering communication channels from a random coding perspective, which is weaker than Blackwell’s criterion.

We provide a self-contained introduction to channel orderings in information theory. Such orderings are a well-studied subject in network information theory [69]. For instance, the capacity region of broadcast channels (without feedback) depends only on the component channels and is known for a number of special cases when one of the components is “better” than the other in some well-defined sense (see, e.g., [70,71]).

**The Blackwell order.** The Blackwell order evaluates channels with a common input alphabet by comparing the minimal expected loss a rational agent incurs when making decisions based on their outputs. This concept is formalized through decision problems under uncertainty (see [24] for an in-depth discussion).

Consider a *decision problem* $\left(\pi_{S},A,\ell \right)$, where A is the set of possible actions, ℓ(s,a) represents the bounded loss incurred when the agent chooses action a∈A in state s∈S, and $\pi_{S}$ is the prior distribution over the state space S.

The agent observes a random variable *Z* via a channel μ:S→Z before choosing an action. A rational agent selects a strategy ρ∈M(Z;A) to minimize the *expected loss* (or *risk*), defined as follows:

(17) $$\begin{array}{c} R\left(\pi_{S},\mu ,\rho ,\ell \right):=\sum_{s\in S}\pi_{S}\left(s\right)\sum_{a\in A}\rho \circ \mu_{s}\left(a\right)\ell \left(s,a\right). \end{array}$$

The *optimal risk* for channel μ is as follows:

(18) $$\begin{array}{c} R\left(\pi_{S},\mu ,\ell \right):=\min_{\sigma \in A_{\mu }}\sum_{s\in S}\pi_{S}\left(s\right)\sum_{a\in A}\sigma_{s}\left(a\right)\ell \left(s,a\right). \end{array}$$

where $A_{\mu }=\left\{\rho \circ \mu :\rho \in M\left(Z;A\right)\right\}$. Optimal strategies can always be chosen deterministically, so it suffices to consider deterministic strategies.

Now, suppose the agent has access to another random variable *Y* via a second channel κ∈M(S;Y) with the same input alphabet S. The agent will *always* prefer *Z* to *Y* if, for any decision problem, the optimal risk using *Z* is no greater than that using *Y*. This leads to the following definition.

**Definition** **2.** *Given μ∈M(S;Z), κ∈M(S;Y), and a probability distribution $\pi_{S}$ on S such that $P_{SZ}\left(s,z\right)=\pi_{S}\left(s\right)\mu_{s}\left(z\right)$ and $P_{SY}\left(s,y\right)=\pi_{S}\left(s\right)\kappa_{s}\left(y\right)$, we say that Z is* always more informative *about S than Y and write $Z{⊒}_{S}Y$ if $R\left(\pi_{S},\kappa ,\ell \right)\geq R\left(\pi_{S},\mu ,\ell \right)$ for any decision problem (with $\pi_{S}$ fixed as above).*

The variables can also be ranked probabilistically: *Z* is *always* preferred over *Y* if, given access to *Z*, a single use of *Y* can be simulated by sampling $y^{\prime }\in Y$ after each observation z∈Z. This implies that *Y* provides no additional utility beyond what *Z* already offers.

**Definition** **3.** *Write $Z{⊒}_{S}^{\prime }Y$ if there exists a random variable $Y^{\prime }$ such that the pairs (S,Y) and $\left(S,Y^{\prime }\right)$ are statistically indistinguishable, and $S-Z-Y^{\prime }$ is a Markov chain.*

Intuitively, *Z* knows everything that *Y* knows about *S* in both these situations. Blackwell showed the equivalence of these two relations [61]. The following is a statement of Blackwell’s theorem for random variables [62]:

**Theorem** **1** (Blackwell’s theorem for random variables)**.** *$Z{⊒}_{S}Y$ *⇔* $Z{⊒}_{S}^{\prime }Y$.*

The original statement of Blackwell’s theorem [61] allows us to directly compare the channels κ and μ and the input distribution on S can be arbitrary.

**Definition** **4.** *We say that μ is* always more informative *than κ and write $\mu {⊒}_{S}\kappa$ if $R\left(\pi_{S},\kappa ,\ell \right)\geq R\left(\pi_{S},\mu ,\ell \right)$ for any $\left(\pi_{S},A,\ell \right)$.*

**Definition** **5.** *We say that *κ is output-degraded *(or* post-garbled) from μ* and write $\mu {⊒}_{S}^{\mathrm{odeg}}\kappa$ if κ=λ∘μ for some λ∈M(Z;Y).*

The relation ${⊒}_{S}^{\mathrm{odeg}}$ is also called the *degradation order* (see, e.g., [72]).

**Theorem 2 (Blackwell’s Theorem** (1953) [61])**.** *$\mu {⊒}_{S}\kappa$ *⇔* $\mu {⊒}_{S}^{\mathrm{odeg}}\kappa$.*

See [73] for a simple proof of Blackwell’s theorem.

If $\pi_{S}$ has full support, then $\mu {⊒}_{S}\kappa \Leftrightarrow Z{⊒}_{S}Y$ (Theorem 4 in [62]) and it suffices to look only at different loss functions. In the sequel, we assume that $\pi_{S}$ has full support, and we call ${⊒}_{S}$ and ${⊒}_{S}$ the *Blackwell orders*.

Strictly speaking, the Blackwell order is only a preorder rather than a partial order as there exist channels κ≠μ that satisfy $\kappa {⊒}_{S}\mu {⊒}_{S}\kappa$ (when κ arises from μ by permuting the output alphabet). However, for our purposes, such channels can be treated as equivalent. We write $\mu {⊐}_{S}\kappa$ if $\mu {⊒}_{S}\kappa$ and $\kappa {⋣}_{S}\mu$. By Blackwell’s theorem, this indicates that μ performs at least as well as κ in any decision problem and that there exist decision problems in which μ outperforms κ.

A related order is the *zonotope order*, which is weaker than the Blackwell order [62,74]. For the special case of binary-valued channel inputs, i.e., |S|=2, the Blackwell order defines a lattice and is identical to the zonotope order [62,74] and its generalization, the *k*-decision order [61].

**The Shannon order.** Shannon proposed a criterion for simulating one channel from another based on a random coding argument [68]. Shannon’s criterion allows for randomization at *both* the input and the output of the simulating channel as well as for shared randomness between its input and output.

**Definition** **6** ([68])**.** *Given two channels $\kappa \in M(S^{\prime };Y)$ and μ∈M(S;Z), we say that μ* includes *κ and write $\mu {⊒}^{inc}\kappa$ if for some k∈N, there exists a probability distribution $g\in P_{\left[k\right]}$ and k pairs of pre- and post-channels $\left(\alpha_{i},\beta_{i}\right)\in M\left(S^{\prime };S\right)\times M\left(Z;Y\right)$, 1≤i≤k, such that $\kappa =\sum_{i=1}^{k}g\left(i\right)\left(\beta_{i}\circ \mu \circ \alpha_{i}\right)$.*

Shannon showed that if $\mu {⊒}^{inc}\kappa$, then the existence of a good coding scheme for κ implies the existence of a good coding scheme for μ, where “goodness” is measured in the sense of low probability of error. Let Σ be the set of all convex combinations of products of the channels in $M(S^{\prime };S)$ with those in M(Z;Y), i.e.,

(19) $$\begin{array}{c} \Sigma =\mathrm{conv}\left(\alpha ⊗\beta \in M\left(S^{\prime }\times Z;S\times Y\right):\alpha \in M\left(S^{\prime };S\right),\;\beta \in M\left(Z;Y\right)\right), \end{array}$$

where conv(C) denotes the convex hull of *C*, and ${\left(\alpha ⊗\beta \right)}_{s^{\prime },z}\left(s,y\right)=\alpha_{s^{\prime }}\left(s\right)\beta_{z}\left(y\right)$ for each s∈S, $s^{\prime }\in S^{\prime }$, z∈Z, and y∈Y. By Carathéodory’s theorem [75], any channel χ∈Σ can be represented as a convex combination of at most $|S^{\prime }\times Z\times S\times Y|+1$ product channels. Given μ∈M(S;Z) and χ∈Σ, define the *skew-composition* $\chi \circ_{s}\mu \in M\left(S^{\prime };Y\right)$ of μ with χ as follows: $\chi \circ_{s}\mu \left(y|s^{\prime }\right)=\sum_{s\in S,z\in Z}\chi_{s^{\prime },z}\left(s,y\right)\mu_{s}\left(z\right)$ for all $s^{\prime }\in S^{\prime }$, y∈Y. We then have the following equivalent characterization of the Shannon order:

**Proposition** **1** ([76])**.** *$\mu {⊒}^{inc}\kappa$ if and only if there exists χ∈Σ such that $\kappa =\chi \circ_{s}\mu$.*

Nasser [76] gave a characterization of the Shannon order that is similar to Blackwell’s theorem.

In Definition 6, the input and output alphabets of both κ and μ may be different. If the channels share a common input alphabet, i.e., $S^{\prime }=S$, then $\mu {⊒}_{S}^{\mathrm{odeg}}\kappa \Rightarrow \mu {⊒}^{inc}\kappa$. The converse implication is not true in general and the Shannon order is weaker than the Blackwell order [25].

**The input-degraded order.** Given two channels that share a common output alphabet, Nasser [77] introduced the following ordering:

**Definition** **7** ([77])**.** *Let $\bar{\kappa }\in M\left(Y;S\right)$ and $\bar{\mu }\in M\left(Z;S\right)$ be two channels with a common output alphabet. We say that* $\bar{\kappa }$ is input-degraded from $\bar{\mu }$* and write $\bar{\mu }{⊒}_{S}^{\mathrm{ideg}}\bar{\kappa }$ if $\bar{\kappa }=\bar{\mu }\circ \bar{\lambda }$ for some $\bar{\lambda }\in M\left(Y;Z\right)$.*

**Proposition** **2** ([77])**.**

$$\begin{array}{c} \bar{\mu }{⊒}_{S}^{\mathrm{ideg}}\bar{\kappa }\Leftrightarrow \mathrm{conv}\left({\left\{{\bar{\kappa }}_{y}\right\}}_{y\in Y}\right)\subset \mathrm{conv}\left({\left\{{\bar{\mu }}_{z}\right\}}_{z\in Z}\right) \end{array}$$

*where conv(C) denotes the convex hull of C.*

Nasser [77] gave a characterization of the input-degraded order that is similar to Blackwell’s theorem.

**The more capable and less noisy orders.** Given two channels κ∈M(S;Y) and μ∈M(S;Z) with a common input alphabet, Körner and Marton introduced the following two orderings [63]:

**Definition** **8.** *μ is said to be* more capable *than κ, denoted $\mu {⊒}^{\mathrm{mc}}\kappa$, if I(S;Z)≥I(S;Y) for every probability distribution $P_{S}\in P_{S}$.*

**Definition** **9.** *μ is said to be* less noisy *than κ, denoted $\mu {⊒}^{\ln}\kappa$, if I(U;Z)≥I(U;Y) for every $P_{US}$ such that U−S−YZ is a Markov chain.*

An equivalent characterization of the less noisy relation is the following [78]: $\mu {⊒}^{\ln}\kappa$ if and only if I(S;Z)−I(S;Y) is a concave function of the input probability distribution $P_{S}$.

We note the following relationship between the Blackwell, less noisy and the more capable preorders:

**Proposition** **3** ([63])**.**

(20) $$\begin{array}{c} \mu {⊒}_{S}^{\mathrm{odeg}}\kappa \Rightarrow \mu {⊒}^{\ln}\kappa \Rightarrow \mu {⊒}^{\mathrm{mc}}\kappa . \end{array}$$

As the following examples show, the converse of neither implication is true in general [63].

**Example** **2** (Broadcast channel consisting of a BSC and a BEC [69,79])**.** *A memoryless broadcast channel model $\left(S,\xi_{s}\left(y,z\right),Y\times Z\right)$ consists of three sets S, Y, and Z, and a channel ξ∈M(S;Y×Z). Let $\kappa_{s}\left(y\right):=\sum_{z\in Z}\xi_{s}\left(y,z\right)$ and $\mu_{s}\left(z\right):=\sum_{y\in Y}\xi_{s}\left(y,z\right)$ be the two components of ξ.*

*Consider a broadcast channel with κ=BSC(p) with crossover probability p∈(0,12), and μ=BEC(ϵ) with erasure probability ϵ∈(0,1). Then, the following hold:*

1. *For 0<ϵ≤2p, Y is output-degraded from Z.*
2. *For 2p<ϵ≤4p(1−p), Z is less noisy than Y, but Y is not output-degraded from Z.*
3. *For 4p(1−p)<ϵ≤h(p), Z is more capable than Y, but not less noisy.*
4. *For h(p)<ϵ<1, ξ does not belong to any of the three classes.*

**Example 3** (Doubly symmetric binary erasure (DSBE) source [12,80])**.** *A DSBE source with parameters (p,ϵ) is defined as follows: $P_{SYZ}\left(s,y,z\right)=P_{SY}\left(s,y\right)p_{Z|SY}\left(z|s,y\right)$ where $P_{SY}\left(0,0\right)=P_{SY}\left(1,1\right)=p/2$, $P_{SY}\left(0,1\right)=P_{SY}\left(1,0\right)=\left(1-p\right)/2$, and $P_{Z|SY}\left(z|s,y\right)$ is an erasure channel, i.e., Z=SY with probability 1−ϵ and Z=e with probability ϵ. Without loss of generality, we may assume $p>\frac{1}{2}$. Then, the following hold:*

1. *For 0<ϵ≤2(1−p), Y is output-degraded from Z.*
2. *For 2(1−p)<ϵ≤4p(1−p), Z is less noisy than Y, but Y is not output-degraded from Z.*
3. *For 4p(1−p)<ϵ≤h(p), Z is more capable than Y, but not less noisy.*
4. *For h(p)<ϵ<1, a DSBE(p,ϵ) source does not belong to any of the three classes.*

## 4. Unique Information and Channel Deficiencies

**Figure 2:**
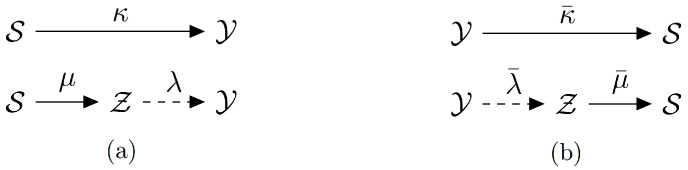
(**a**) Simulation of the channel κ through a randomization at the output of μ, where κ and μ share a *common input* alphabet S. (**b**) Simulation of the channel $\bar{\kappa }$ through a randomization at the input of $\bar{\mu }$, where $\bar{\kappa }$ and $\bar{\mu }$ share a *common output* alphabet S.

How can we determine whether *Y* possesses unique information about *S* that is not available to *Z*? Consider the channels κ and μ with a *common input* alphabet S, as illustrated in Figure 2a. If μ can be reduced to κ by appending a post-channel λ at its *output*, then μ can be said to *include* κ. Similarly, for the channels $\bar{\kappa }$ and $\bar{\mu }$ with a *common output* alphabet S, as shown in Figure 2b, $\bar{\mu }$ can be considered to include $\bar{\kappa }$ if it reduces to $\bar{\kappa }$ by adding a pre-channel $\bar{\lambda }$ at its *input*.

In both cases, one would expect *Y* to provide no unique information about *S* relative to *Z*. A nonzero unique information would then serve as a measure of the extent to which one channel deviates from being an inclusion or randomization of the other.

The function UI in Definition 1 is based on the idea of approximating one channel by randomizing its *output* (see Figure 2a). In contrast, Harder et al. [26] defined a measure of shared information through a difference in two KL divergence terms, where one term involves randomization at the *input* (see Figure 2b). In both cases, the resulting decompositions of the total mutual information are non-negative.

Banerjee et al. [16] introduce two quantities that generalize Le Cam’s notion of *weighted deficiency* [23,24,25] between channels. Weighted deficiencies quantify the cost of approximating one channel from another via randomizations and are closely related to the function UI. Depending on whether the randomization occurs at the output or input, two different forms of weighted deficiency arise: the *weighted output KL deficiency* and the *weighted input KL deficiency*. Both of these induce non-negative bivariate decompositions [16]. Interestingly, the decomposition corresponding to the weighted input deficiency coincides with the one introduced by Harder et al. [26] (see Proposition 8).

### 4.1. Generalized Le Cam Deficiencies

The Blackwell order provides a natural criterion to determine if a variable *Y* has unique information about *S* with respect to *Z* or not; see Definitions 2 and 3.

**Definition** **10** (Blackwell property)**.** *Y* has no unique information *about S with respect to Z*:⟺* $Z{⊒}_{S}^{\prime }Y$.*

The function UI satisfies the Blackwell property (see Lemma 1). When UI(S;Y\Z) vanishes, we say that *Z* is *Blackwell-sufficient* for *Y* with respect to *S*.

Theorem 1 states that if the relation $Z{⊒}_{S}Y$ (resp. $Y{⊒}_{S}Z$) does not hold, then there exist a loss function and a set of actions that render *Y* (resp. *Z*) more useful. This statement motivates the following definition [6]:

**Definition** **11.** *Y* has unique information *about S with respect to Z if there exists a set of actions A and a loss function $\ell \left(s,a\right)\in R^{S\times A}$ such that $R\left(\pi_{S},\kappa ,\ell \right)<R\left(\pi_{S},\mu ,\ell \right)$.*

The relation ${⊒}_{S}^{\mathrm{odeg}}$ is a preorder on the family of all channels with the same input alphabet S (see Definition 5). In general, we cannot always simulate one channel by a randomization of the other. To be able to compare any two channels, Lucien Le Cam introduced the notion of channel *deficiencies* [23,24]:

**Definition** **12.** *Given μ∈M(S;Z) and κ∈M(S;Y), the* Le Cam deficiency of μ with respect to κ *is*

(21) $$\begin{array}{c} \delta \left(\mu ,\kappa \right):=\underset{\lambda \in M(Z;Y)}{\inf}\underset{s\in S}{\sup}{∥\lambda \circ \mu_{s}-\kappa_{s}∥}_{\mathrm{TV}}. \end{array}$$

*where ${∥\cdot ∥}_{\mathrm{TV}}$ denotes the total variation distance.*

Note that δ(μ,κ)=0 if and only if $\mu {⊒}_{S}^{\mathrm{odeg}}\kappa$.

**Definition** **13.** *Given μ∈M(S;Z), κ∈M(S;Y) and a probability distribution $\pi_{S}$ on S, the* weighted Le Cam deficiency of μ with respect to κ *is*

(22) $$\begin{array}{c} \delta^{\pi }\left(\mu ,\kappa \right):=\underset{\lambda \in M(Z;Y)}{\inf}E_{s∼\pi_{S}}{∥\lambda \circ \mu_{s}-\kappa_{s}∥}_{\mathrm{TV}}. \end{array}$$

The *Le Cam randomization criterion* [23] establishes that deficiencies quantify the maximal gap in optimal risks between decision problems when using the channel μ instead of κ.

**Theorem** **3** ([23])**.** *Fix μ∈M(S;Z), κ∈M(S;Y), and a probability distribution $\pi_{S}$ on S, and write ${∥\ell ∥}_{\infty }=\max_{s,a}\ell \left(s,a\right)$. For every ϵ>0, $\delta^{\pi }\left(\mu ,\kappa \right)\leq \epsilon$ if and only if $R\left(\pi_{S},\mu ,\ell \right)-R\left(\pi_{S},\kappa ,\ell \right)\leq \epsilon {∥\ell ∥}_{\infty }$ for any set of actions A and any bounded loss function ℓ.*

Raginsky [25] introduced a broad class of deficiency-like quantities based on a “generalized” divergence between probability distributions that maintains a monotonicity property with respect to data processing. Specializing this to the KL divergence, we have the following definition:

**Definition** **14.** *The* output KL deficiency of μ with respect to κ *is*

(23) $$\begin{array}{c} \delta_{o}\left(\mu ,\kappa \right):=\underset{\lambda \in M(Z;Y)}{\inf}\underset{s\in S}{\sup}D\left(\kappa_{s}∥\lambda \circ \mu_{s}\right), \end{array}$$

*where the subscript o in $\delta_{o}$ emphasizes the fact that the randomization is at the* output *of the channel μ.*

In a spirit similar to [25] and Section 6.2 in [24], one can define a weighted output KL deficiency [16]:

**Definition** **15.** *The* weighted output KL deficiency of μ with respect to κ *is*

(24) $$\begin{array}{c} \delta_{o}^{\pi }\left(\mu ,\kappa \right):=\min_{\lambda \in M(Z;Y)}D(\kappa ∥\lambda \circ \mu |\pi_{S}). \end{array}$$

The weighted output KL deficiency quantifies the cost of approximating one observed variable from the other (and vice versa) through Markov kernels. Notably, $\delta_{o}^{\pi }\left(\mu ,\kappa \right)=0$ if and only if $Z{⊒}_{S}^{\prime }Y$, capturing the intuition that a small value of $\delta_{o}^{\pi }\left(\mu ,\kappa \right)$ implies that *Z* is *approximately Blackwell-sufficient* for *Y* with respect to *S*. Using Pinsker’s inequality, we obtain the following:

(25) $$\begin{array}{c} \delta^{\pi }\left(\mu ,\kappa \right)\leq \sqrt{\frac{\ln\left(2\right)}{2}\delta_{o}^{\pi }\left(\mu ,\kappa \right)}. \end{array}$$

Bounding the weighted output KL deficiency is sufficient to guarantee that the differences in optimal risks remain bounded for any decision problem of interest [16]:

**Proposition** **4.** *Fix μ∈M(S;Z), κ∈M(S;Y), and a prior probability distribution $\pi_{S}$ on S, and write ${∥\ell ∥}_{\infty }=\max_{s,a}\ell \left(s,a\right)$. For every ϵ>0, if $\delta_{o}^{\pi }\left(\mu ,\kappa \right)\leq \epsilon$, then $R\left(\pi_{S},\mu ,\ell \right)-R\left(\pi_{S},\kappa ,\ell \right)\leq \sqrt{\epsilon \frac{\ln\left(2\right)}{2}}{∥\ell ∥}_{\infty }$ for any set of actions A and any bounded loss function ℓ.*

Recall the data processing inequality for the mutual information:

(26) $$\begin{array}{c} Z-Y-W\Rightarrow I(Z;W)\leq \min\{I(Z;Y),I(Y;W)\}. \end{array}$$

Lemma 4 shows that the weighted output KL deficiency satisfies a similar inequality:

**Lemma** **4.** *Let μ∈M(S;Z), κ∈M(S;Y), and ν∈M(S;W) be three channels with a common input alphabet and let $\pi_{S}$ be a given distribution on S. Then,*

$$\begin{array}{c} Z{⊒}_{S}Y{⊒}_{S}W\Rightarrow \delta_{o}^{\pi }\left(\mu ,\nu \right)\leq \min\left\{\delta_{o}^{\pi }\left(\mu ,\kappa \right),\delta_{o}^{\pi }\left(\kappa ,\nu \right)\right\}. \end{array}$$

See Appendix B for a proof. One can also define a weighted deficiency for the input-degraded order in Definition 7 [16].

**Definition** **16.** *The* weighted input KL deficiency of $\bar{\mu }$ with respect to $\bar{\kappa }$ *is*

(27) $$\begin{array}{c} \delta_{i}^{\pi }\left(\bar{\mu },\bar{\kappa }\right):=\min_{\bar{\lambda }\in M\left(Y;Z\right)}D(\bar{\kappa }∥\bar{\mu }\circ \bar{\lambda }\left|\pi_{Y}\right), \end{array}$$

*where the subscript i in $\delta_{i}$ emphasizes the fact that the randomization is at the* input *of the channel $\bar{\mu }$.*

The weighted input KL deficiency satisfies the following monotonicity property:

**Lemma** **5.** *Let $\bar{\mu }\in M\left(Z;S\right)$, $\bar{\kappa }\in M\left(Y;S\right)$, and $\bar{\nu }\in M\left(W,S\right)$ be three channels with a common output alphabet, and let $\pi_{W}$ be a given distribution on W. Then,*

$$\begin{array}{c} \bar{\mu }{⊒}_{S}^{\mathrm{ideg}}\bar{\kappa }\Rightarrow \delta_{i}^{\pi }\left(\bar{\mu },\bar{\nu }\right)\leq \delta_{i}^{\pi }\left(\bar{\kappa },\bar{\nu }\right). \end{array}$$

The proof is similar to the first part of the proof of Lemma 4 and is omitted.

### 4.2. Non-Negative Mutual Information Decompositions

Given an information measure that captures some aspect of unique information but does not satisfy the consistency condition (7), we can construct the corresponding bivariate information decomposition as follows:

**Lemma** **6** ([81], Proposition 9)**.** *Let $\delta :P_{S\times Y\times Z}\to R$ be a non-negative function that satisfies*

$$\begin{array}{c} \delta (S;Y\backslash Z)\leq \min\{I(S;Y),I(S;Y|Z)\}. \end{array}$$

*Then, a bivariate information decomposition is given by*

$$\begin{array}{c} \begin{array}{cc} UI_{\delta }\left(S;Y\backslash Z\right) & =\max\left\{\delta (S;Y\backslash Z),\delta (S;Z\backslash Y)+I(S;Y)-I(S;Z)\right\},\\ UI_{\delta }\left(S;Z\backslash Y\right) & =\max\left\{\delta (S;Z\backslash Y),\delta (S;Y\backslash Z)+I(S;Z)-I(S;Y)\right\},\\ SI_{\delta }\left(S;Z,Y\right) & =\min\left\{I(S;Y)-\delta (S;Y\backslash Z),I(S;Z)-\delta (S;Z\backslash Y)\right\},\\ CI_{\delta }\left(S;Z,Y\right) & =\min\left\{I(S;Y|Z)-\delta (S;Y\backslash Z),I(S;Z|Y)-\delta (S;Z\backslash Y)\right\}. \end{array} \end{array}$$

We refer to the construction in Lemma 6 as the *UI construction*. The unique information $UI_{\delta }$ generated by this construction is the smallest UI function among all bivariate information decompositions with UI≥δ.

This construction can be used to derive new non-negative bivariate decompositions.

#### 4.2.1. Decomposition Based on the Weighted Output KL Deficiency

**Proposition** **5** ([16])**.** *Let (S,Y,Z)∼P, and let $\pi_{S}$ be the marginal distribution of S. Let κ∈M(S;Y) resp. μ∈M(S;Z) be two channels describing the conditional distribution of Y resp. Z, given S. Define*

(28) $$\begin{array}{c} \delta \left(S;Y\backslash Z\right)=\delta_{o}^{\pi }\left(\mu ,\kappa \right), \end{array}$$

*where $\delta_{o}$ is the weighted output KL deficiency *(24)*. Then, the functions $UI_{\delta }$, $SI_{\delta }$, and $CI_{\delta }$ in Lemma 6 define a non-negative bivariate decomposition.*

**Lemma** **7** ([16])**.** *Define*

(29) $$\begin{array}{c} UI_{o}\left(S;Y\backslash Z\right)=\max\left\{\delta_{o}^{\pi }\left(\mu ,\kappa \right),\delta_{o}^{\pi }\left(\kappa ,\mu \right)+I\left(S;Y\right)-I\left(S;Z\right)\right\}. \end{array}$$

*Then, $UI_{o}\left(S;Y\backslash Z\right)$ vanishes if and only if Y has no unique information about S with respect to Z (according to Definition 10).*

From Lemma 3, we have the following relationship between the different quantities:

**Lemma** **8.**
$$\begin{array}{cc} \delta_{o}^{\pi }\left(\mu ,\kappa \right) & \leq UI_{o}\left(S;Y\backslash Z\right)\leq UI\left(S;Y\backslash Z\right), \end{array}$$

The next proposition follows from Lemmas 1 and 7, and Definition 15.

**Proposition** **6.**
$$\begin{array}{c} \delta_{o}^{\pi }\left(\mu ,\kappa \right)=0\Leftrightarrow UI_{o}\left(S;Y\backslash Z\right)=0\Leftrightarrow UI\left(S;Y\backslash Z\right)=0. \end{array}$$

#### 4.2.2. Decomposition Based on the Weighted Input KL Deficiency

**Proposition** **7** ([16])**.** *Let (S,Y,Z)∼P, and let $\pi_{Y}$ resp. $\pi_{Z}$ be the induced marginal distributions of Y resp. Z, both assumed to have full support. Let $\bar{\kappa }\in M\left(Y;S\right)$ and $\bar{\mu }\in M\left(Z;S\right)$ be two channels such that $\bar{\kappa }=P_{S|Y}$ and $\bar{\mu }=P_{S|Z}$. Define*

(30) $$\begin{array}{c} \delta \left(S;Y\backslash Z\right)=\delta_{i}^{\pi }\left(\bar{\mu },\bar{\kappa }\right), \end{array}$$

*where $\delta_{i}$ is the weighted input KL deficiency *(27)*. Then, the functions $UI_{\delta }$, $SI_{\delta }$, and $CI_{\delta }$ in Lemma 6 define a non-negative bivariate decomposition.*

Harder et al. [26] introduced a measure of *shared information* based on reverse information (rI) projections [82] onto a convex set of probability measures.

**Definition** **17.** *For $C\subset P_{S}$, let conv(C) denote the convex hull of C. Let*

$$Q_{y↘Z}\left(S\right)\in \underset{Q\in \mathrm{conv}\left({\left\{{\bar{\mu }}_{z}\right\}}_{z\in Z}\right)\subset P_{S}}{arg min}D\left({\bar{\kappa }}_{y}∥Q\right)$$

*be the *rI-projection *of ${\bar{\kappa }}_{y}$ onto the convex hull of the points ${\left\{{\bar{\mu }}_{z}\right\}}_{z\in Z}\in P_{S}$. Define the* projected information of *Y* onto *Z* with respect to S *as*

(31) $$\begin{array}{c} I_{S}\left(Y↘Z\right):=E_{\left(s,y\right)∼\bar{\kappa }\times \pi_{Y}}\log\frac{Q_{y↘Z}\left(s\right)}{\bar{\kappa }\circ \pi_{Y}\left(s\right)}, \end{array}$$

*and the shared information as*

(32) $$\begin{array}{c} SI_{red}\left(S;Y,Z\right):=\min\left\{I_{S}\left(Y↘Z\right),I_{S}\left(Z↘Y\right)\right\}. \end{array}$$

Proposition 8 states that implicit in the above construction is the weighted input KL deficiency $\delta_{i}^{\pi }\left(\bar{\mu },\bar{\kappa }\right)$.

**Proposition** **8** ([16])**.** *$I_{S}\left(Y↘Z\right)=I\left(S;Y\right)-\delta_{i}^{\pi }\left(\bar{\mu },\bar{\kappa }\right)$.*

An immediate consequence of Proposition 8 is that the decomposition proposed by Harder et al. [26] and that in Proposition 7 are equivalent.

**Remark** **1** ($SI_{red}$ is not continuous)**.** *$I_{S}\left(Y↘Z\right)$ and $I_{S}\left(Z↘Y\right)$ are defined in terms of conditional probability ${\bar{\kappa }}_{y}=P_{S|Y=y}$ and ${\bar{\mu }}_{z}=P_{S|Z=z}$, which are only defined for those y,z with $\pi_{Y}\left(y\right)>0$ and $\pi_{Z}\left(z\right)>0$. Therefore, $I_{S}\left(Y↘Z\right)$ and $I_{S}\left(Z↘Y\right)$ are discontinuous when probabilities tend to zero. For a concrete example, see Example 3 in* [18].

**Remark** **2** (Vanishing sets of UI and deficiencies)**.** *The Blackwell order compares two channels with a* common input *alphabet. This order has found applications in network information theory* [69,79] *. In wiretap channel models* [64,65] *(see Section 5.2), one considers a memoryless broadcast channel ξ:S→Y×Z where Alice selects the inputs to ξ, while Bob and Eve observe, resp., the Y-outputs and the Z-outputs. Bob’s component channel is defined as $\kappa_{s}\left(y\right)=\sum_{z\in Z}\xi_{s}\left(y,z\right)$ and Eve’s as $\mu_{s}\left(z\right)=\sum_{y\in Y}\xi_{s}\left(y,z\right)$. The secrecy capacity of the wiretap channel, $C_{S}$, quantifies a deviation from the less noisy order and depends on ξ only through the component channels κ and μ (see Proposition 9). Likewise, when the distribution of the input to ξ is fixed, the UI and weighted output deficiency $\delta_{o}^{\pi }$ quantify a deviation from the Blackwell order and depend on ξ only through κ and μ. Proposition 6 shows that the sets on which UI and $\delta_{o}^{\pi }$ vanish are the same.*

*On the other hand, the weighted input deficiency $\delta_{i}^{\pi }$ quantifies a deviation from the input-degraded order, which compares two channels with a* common output *alphabet. This ordering appears more natural in some settings, e.g., when learning a classifier (see, e.g.,* [81]*). We can again define a channel model $\bar{\xi }:Y\times Z\to S$. The associated component channels $\bar{\kappa }\left(s|y\right)$ and $\bar{\mu }\left(s|z\right)$ are, however, not uniquely determined by $\bar{\xi }$ (also see Remark 1). In Theorem 22 of* [6]*, it was claimed that the vanishing sets of $\delta_{i}^{\pi }$ and UI coincide. However, Banerjee et al. [83] showed that this assertion is incorrect (see Example 28b in [83]).*

**Remark** **3** (Decompositions based on known bounds on the secret key rates)**.** *In Section 5, we show that the function UI shares conceptual similarities with secret key rates* [12,84]. *The UI construction can be used to obtain bivariate information decompositions from the one-way ($S_{\to }$, *(42)*) and two-way secret key rates ($S_{↔}$), as well as from related information functions defined as bounds on these rates. These functions include the secrecy capacity of the wiretap channel $C_{S}$* (36)*, the* intrinsic information *$I_{↓}$*(47)*, the* reduced intrinsic information *$I_{↓↓}$*(50)*, and the* minimum intrinsic information *$B_{1}$*(52)*. Each of these bounds can be expressed as optimization problems over Markov kernels of bounded size. For the complete chain of inequalities, see *(57)*. Like UI, both $S_{\to }$ and $C_{S}$ depend solely on the marginal distributions of the pairs (S,Y) and (S,Z). However, unlike UI, none of these functions satisfy the consistency condition *(7)*. Nevertheless, since these bounds are upper-bounded by min{I(S;Y),I(S;Y|Z)}, we can utilize the UI construction outlined in Lemma 6 to derive new non-negative decompositions. An analysis of the properties of these decompositions is reserved for future study.*

## 5. Unique Information and Secrecy Monotones

The contents of this section have a distinct cryptographic flavor. Our main goal is to establish the operational significance of the UI in Definition 1. In order to keep the exposition reasonably self-contained, we collect all relevant definitions and models in Section 5.2. Theorem 8, the triangle inequality for the UI (Property P.7), and Theorem 9 are the main results in this section. Theorem 8 was first derived in [16], while the contents of Section 5.4 and Section 5.5 expands on the work in [16,17].

### 5.1. Motivation and Synopsis

Consider the *source model* for secret key agreement between Alice and Bob, who are distant from each other and must communicate over a noiseless but insecure (public) channel in the presence of an adversary, Eve [12,85]. Alice, Bob, and Eve observe i.i.d. copies of random variables *S*, *Y*, and *Z*, respectively, where (S,Y,Z)∼*P*. Alice and Bob aim to agree on a secret key by exchanging messages over the public channel according to a predefined protocol. Eve is aware of the protocol and can intercept and read all the messages exchanged. The maximum rate at which Alice and Bob can compute a key such that Eve’s total information (from both *Z* and the entire communication) about the key is negligibly small is referred to as the *two-way secret key rate*, $S_{↔}$. If Alice is allowed to send only one message and Bob sends none, the corresponding key rate is called the *one-way secret key rate*, $S_{\to }$.

The secret key rates are conceptually similar to the function UI. While UI(S;Y\Z) is interpreted as the *information about S known to Y, but not to Z*, $S_{↔}\;\left(S;Y\;\left|Z\right.\right)$ can be interpreted as the *information common to S and Y, which is unique with respect to Z*.

For example, consider the Rdn distribution from Example 1, where Alice, Bob, and Eve each share one uniformly random bit. In this case, since Eve knows the exact values of *S* and *Y*, Alice and Bob cannot share a secret. This is reflected in the values of UI(S;Y\Z) and UI(Y;S\Z), both of which are zero.

As another example, consider the Xor distribution in Example 1, where the values of any two variables in (S,Y,Z) determine the third. Clearly, if Alice can only observe *S* and Bob can only observe *Y*, they cannot generate a secret key. This is also apparent from the values of UI(S;Y\Z) and UI(Y;S\Z), both of which are zero. However, if Alice is also able to observe *Z*, she can compute *Y*, which can then be used as a key that is perfectly secret from Eve, since Eve’s variable *Z* is independent of the key *Y*.

Intuitively, when Alice and Bob share some common information that is unique with respect to Eve, they can *exploit* this information to generate a secret key. A distribution combining elements of the Xor and Rdn models exemplifies the potential advantage of such a setup:

**Example** **4** (TheXorRdn distribution [12,84])**.** *Consider the following distribution: $P_{SYZ}\left(0,0,0\right)$ = $P_{SYZ}\left(0,1,1\right)=P_{SYZ}\left(1,0,1\right)=P_{SYZ}\left(1,1,0\right)=\frac{1}{8}$, and $P_{SYZ}\left(2,2,2\right)=P_{SYZ}\left(3,3,3\right)=\frac{1}{4}$. The table below shows the distribution (with Z’s value in parentheses):*

*S*
*Y* (*Z*)0123018 (0)18 (1)..118 (1)18 (0)..2..14 (2).3...14 (3)

*If Eve observes *2* or *3*, she can determine the exact values of S and Y. When she observes *0* or *1*, she can infer that Alice and Bob’s values lie within {0,1}, but within this range, their observations are independent. Consequently, no secret key agreement is possible in this case. This is reflected in the values of UI(S;Y\Z) and UI(Y;S\Z), both of which are zero.*

*Consider now the modified distribution: $P_{SYZ}\left(0,0,0\right)=P_{SYZ}\left(0,1,1\right)=P_{SYZ}\left(1,0,1\right)=P_{SYZ}\left(1,1,0\right)=\frac{1}{8}$, and $P_{SYZ}\left(2,2,0\right)=P_{SYZ}\left(3,3,1\right)=\frac{1}{4}$, where Eve’s variable Z can only assume binary values.*

*S*
*Y* (*Z*)0123018 (0)18 (1)..118 (1)18 (0)..2..14 (0).3...14 (1)

*Now Bob (resp. Alice) has 1 bit of unique information about Alice’s (resp. Bob’s) values with respect to Eve (namely, the ability to distinguish whether Alice sees values in the* XOR *or the* RDN *quadrant) which can be used to agree on 1 bit of secret.*

A computable characterization of the one-way secret key rate is known [66] (see Theorem 6). In contrast, determining the two-way key rate for a given distribution, or even the condition when it is positive, seems difficult, and its value is known only for a handful of distributions [66,67,86,87]. For protocols with unbounded communication, computing the two-way key rate for a general distribution is a fundamental and open area of inquiry in information-theoretic cryptography.

A standard technique for deriving upper bounds on the two-way key rate is to consider functions of joint distributions called *secrecy monotones* or simply *monotones*, which satisfy the following property: In any secret key agreement protocol, a monotone can *never increase* if Alice and Bob are only allowed to perform a well-defined class of physical operations called local operations (LOs) and public communication (PC) [14,67,88]. Theorem 8 shows that the UI is an upper bound on the one-way secret key rate. This is a consequence of the fact that the function UI is a monotone when the class of allowed operations is local operations and one-way public communication.

The state-of-the-art upper bounds on the two-way key rate are based on the following key property (see Theorem 4 in [67]): For any tuple $\left(S,Y,Z,Z^{\prime }\right)$,

(33) $$\begin{array}{cc} S_{↔}\;\left(S;Y\;\left|Z\right.\right) & \leq S_{↔}\;\left(S;Y\;\left|Z^{\prime }\right.\right)+S_{\to }\;\left(SY;Z^{\prime }\;\left|Z\right.\right). \end{array}$$

In [67,89], a heuristic interpretation of this decomposition is provided: Let $s=S_{↔}\;\left(S;Y\;\left|Z\right.\right)$. Consider a fourth party, Charlie, who receives i.i.d. copies of $Z^{\prime }$ but does not have access to the public channel. If we decompose *s* into two parts: $s_{1}$, which Charlie does not know, and $s_{2}=s-s_{1}$, which Charlie knows about the shared secret key between *S* and *Y* with respect to *Z*, then $s_{1}$ is at most $S_{↔}\;\left(S;Y\;\left|Z^{\prime }\right.\right)$, while $s_{2}$ is at most $S_{\to }\;\left(SY;Z^{\prime }\;\left|Z\right.\right)$.

Gohari et al. [80] gave an alternative interpretation of (33): For any $\left(S,Y,Z,Z^{\prime }\right)$∼*P*, if the induced channel $P_{Z|SY}$ dominates $P_{Z^{\prime }|SY}$ in the *less noisy* sense (see Definition 9), then the second term $S_{\to }\;\left(SY;Z^{\prime }\;\left|Z\right.\right)$ vanishes. Thus, $S_{\to }\;\left(SY;Z^{\prime }\;\left|Z\right.\right)$ represents the “penalty” for deviating from the less noisy condition when substituting $P_{Z|SY}$ with $P_{Z^{\prime }|SY}$.

The function UI satisfies a triangle inequality, which implies the following property that resembles (33): For any $\left(S,Y,Z,Z^{\prime }\right)$,

(34) $$\begin{array}{c} UI\left(S;Y\backslash Z\right)\leq UI\left(S;Y\backslash Z^{\prime }\right)+UI\left(SY;Z^{\prime }\backslash Z\right). \end{array}$$

From (34), we conclude that the UI is never greater than the best-known computable upper bound on $S_{↔}$. We also give an example where the UI is not lower than the best-known lower bound on the two-way rate. We conjecture that the UI lower-bounds the two-way key rate and discuss implications of the conjecture.

### 5.2. Information-Theoretic Secrecy Models

We begin by reviewing some fundamental models in information-theoretic cryptography. Some excellent references include [13,14,15] and Section 17.3 in [27].

Suppose that Alice wishes to transmit a message to Bob over a *noiseless* channel such that an adversary, Eve, who has access to the channel, obtains no information about the message. The channel is assumed to be *authenticated* in the sense that Eve has only read access to the channel and cannot modify or insert messages without being detected. Authentication can be guaranteed, for instance, if Alice and Bob initially share a short secret key [90]. The assumption that the channel is noiseless entails no loss of practicality if we assume that powerful error correction schemes exist, so that the message can be recovered with an arbitrarily small probability of error. This assumption is convenient because it allows us to focus solely on secrecy without having to worry about communication efficiency. We will call such a noiseless and authenticated channel the *public channel*. The terminology is, of course, suggestive of the fact that the channel is insecure. While it is often impractical to assume that a secure channel (e.g., a trusted courier) is always available whenever such a need arises, without loss of generality, we will assume that insecure public channels (e.g., telephone lines) are always available.

**The Shannon model.** Shannon introduced a simple model of a cryptosystem [91] as follows. Let random variables M∈M and C∈C model, resp., the message and the codeword or ciphertext. Alice and Bob share a common secret key modeled by a random variable K∈K. We assume that *K* is independent of *M*. Let e:M×K→C and d:C×K→M denote, resp., Alice’s encoding and Bob’s decoding function. The pair (e,d) is called a *coding scheme*. We assume that Eve has no knowledge of the key but knows the coding scheme and that Bob can decode messages without error, i.e., M=d(C,K) if C=e(M,K). Alice encodes *M* into a ciphertext *C* using the secret key before sending it over the public channel. Since the channel is public, Eve receives an identical copy of *C* as Bob. A coding scheme is said to achieve *perfect secrecy* if Eve’s equivocation about the message given the ciphertext as measured by the conditional entropy H(M|C) equals her a priori uncertainty about the message, i.e., H(M|C)=H(M), or, equivalently I(M;C)=0. Shannon gave a necessary condition for communication in perfect secrecy.

**Theorem** **4** ([91])**.** *If a coding scheme achieves perfect secrecy, then H(K)≥H(M).*

To see this, note that by assumption, H(M)=H(M|C). Since Bob can decode messages without error, we have H(M|CK)=0. The claim follows from H(M)=H(M|C)=H(M|C)−H(M|CK)=I(K;M|C)≤H(K|C)≤H(K).

From an algorithmic perspective, perfect secrecy can be realized using a public channel and a secret key by means of a simple coding scheme called the *one-time pad* (OTP) [92]:

**Example** **5** (OTP)**.** *The message M is a l-bit string and the key K is a uniformly distributed l-bit string which is independent of the message. Alice computes C=M⊕K and Bob computes C⊕K, where *⊕* denotes a bit-wise *Xor* operation. Alice’s encoding guarantees that H(C)=l. Also, H(C|M)=H(K|M)=H(K)=l, since there is a one-to-one mapping between C and K given M, and K is independent of M. We thus have I(M;C)=H(C)−H(C|M)=0, which shows that the OTP achieves perfect secrecy.*

The OTP guarantees that Eve can do no better than randomly guess *M* and that there exists *no* algorithm that could extract any information about *M* from *C*. The OTP is *unconditionally secure* in the sense that this is true even when Eve has unlimited computing power. The OTP is also provably secure in the sense that very precise statements can be made about the information that is leaked to Eve under some well-defined notion of statistical independence (or near-independence) of the message from Eve’s observations.

Contrast this with *computationally secure* cryptosystems which are based on computational complexity theory [93,94]. The security of these systems is based on the following assumptions: (a) Eve’s computational resources, specified by some model of computation, are bounded and, (b) certain one-way functions exist that are computationally “hard” to invert (see Chapter 2 in [95]). The existence of such one-way functions is an open conjecture [96]. Candidates for one-way functions are the discrete-logarithm and the integer factorization problem which form, resp., the basis of the Diffie–Hellman key exchange [93] and the RSA public-key cryptosystem [94]. Efficient randomized algorithms are known for the discrete-logarithm and the integer factorization problem on quantum computers [97]. Hence, public-key cryptosystems are not only provably insecure in theory, but also potentially in practice.

The OTP implements unconditional secrecy with low complexity. However, its applicability is limited in practice since Alice and Bob must share a secret key in advance. Furthermore, the key must at least be as long as the message and can be used only once. Theorem 4, however, shows that the OTP is optimal with respect to key length. Hence, any unconditionally secure cryptosystem is necessarily as impractical as the OTP.

On the other hand, the assumption that the Eve has precisely the *same* information as Bob (except for the secret key) is unrealistic in general. This is, for instance, the case in computational security schemes, which assume that Eve’s channel is noiseless, but her computational resources are bounded. Physical communication channels are noisy, and in real systems, Eve has some minimal uncertainty about the signal received by Bob. The following example shows that if Eve’s observation is in some sense “noisier” than Bob’s, then information-theoretically secure communication is possible even when Alice and Bob do *not* share a secret key in advance.

**Example** **6** (The binary erasure wiretap channel [13,64])**.** *Consider the following simplistic scenario: Alice wishes to send one bit of information to Bob over a binary public channel. Eve’s channel is not as perfect as Bob’s: she observes a corrupted version of the bit at the output of a BEC(ϵ). Hence, Eve knows the bit with probability 1−ϵ, and her equivocation equals ϵ.*

*Let us assume that Alice has access to a source of private randomness which is independent of the message and the channel. To augment Eve’s equivocation, Alice chooses a message M uniformly at random from the set {0,1} and employs the following coding scheme: She takes the set of all n-bit sequences ${\left\{0,1\right\}}^{n}$ and splits them into two* bins*, $b_{0}$ and $b_{1}$, which comprise all n-bit sequences with odd, resp., even parity. To send a message m∈{0,1}, Alice transmits a codeword $S^{n}$ chosen uniformly at random in $b_{m}$. The rate of the code is $\frac{1}{n}$ bits per transmitted channel symbol.*

*Clearly, Bob can recover the correct message by determining the parity of the received codeword. Eve, however, observes a sequence $Z^{n}\in {\left\{0,1,e\right\}}^{n}$ that has nϵ erasures on average. Define a binary random variable E such that E=0 if $Z^{n}$ contains no erasures and E=1 otherwise. If E=0, Eve can decode the message correctly. However, if E=1, the parity of the erased bits is equally likely to be odd or even. We can lower bound Eve’s equivocation as follows:*

$$\begin{array}{cc} H\left(M|Z^{n}\right)\geq H\left(M|Z^{n},E\right) & \overset{\left(a\right)}{=}H\left(M|Z^{n},E=1\right)\left(1-{\left(1-\epsilon \right)}^{n}\right)\\ & =H\left(M\right)\left(1-{\left(1-\epsilon \right)}^{n}\right)=1-{\left(1-\epsilon \right)}^{n}, \end{array}$$

*where equality (a) follows from the fact that $\;Pr\;\left[E=1\right]=1-{\left(1-\epsilon \right)}^{n}$ and $H(M|Z^{n},E=0)=0$. Hence, $I\left(M;Z^{n}\right)\leq {\left(1-\epsilon \right)}^{n}$, which vanishes exponentially fast in n. By repeating this process, Alice and Bob can agree on a secret key of arbitrary length.*

The coding scheme in Example 6 is secure in an asymptotic sense since it requires that the *total* amount of information leaked to Eve vanishes as *n* goes to infinity, i.e., $\lim_{n\to \infty }I\left(M;Z^{n}\right)=0$. This is less stringent than requiring an exact statistical independence of *M* and $Z^{n}$ and is often mathematically more tractable [13]. We call this the *strong secrecy* condition. Alternatively, one can require that the *rate* at which information is leaked to Eve vanishes as *n* goes to infinity, i.e., $\lim_{n\to \infty }\frac{1}{n}I\left(M;Z^{n}\right)=0$. We call this the *weak secrecy* condition. This requirement is weaker than the strong secrecy condition since it is satisfied as long as $I(M;Z^{n})$ grows at most sublinearly in *n*.

**The wiretap channel model.** Example 6 shows that one can use a noisy channel as a “cryptographic resource”. We now consider a more general case first considered by Wyner [64] and subsequently generalized by Csiszár and Körner [65], where the main channel from Alice to Bob is no longer noiseless. Given a broadcast channel ξ∈M(S;Y×Z), let $\kappa_{s}\left(y\right):=\sum_{z\in Z}\xi_{s}\left(y,z\right)$ and $\mu_{s}\left(z\right):=\sum_{y\in Y}\xi_{s}\left(y,z\right)$ be the two components of ξ. Alice chooses the input to ξ, and we refer to κ as the “main channel” and μ as “Eve’s channel”.

Alice uses a stochastic encoder to map the message *M* into an input $S^{n}$ to the channels κ and μ. Bob and Eve observe, resp., the corresponding outputs $Y^{n}$ and $Z^{n}$. Bob wishes to decode the message with a small probability of error such that Eve’s information about the message is arbitrarily small. The largest achievable rate at which Alice can send a message to Bob is called the *secrecy capacity* $C_{S}\left(S;Y|Z\right)$. We give a formal definition.

**Definition** **18** ([65])**.** *The secrecy capacity of the wiretap channel is the largest rate R such that for every ϵ>0, δ>0, and sufficiently large n, there exist random variables M, $S^{n}$, $Y^{n}$, and $Z^{n}$ satisfying $M-S^{n}-Y^{n}Z^{n}$, where $Y^{n}$ and $Z^{n}$ are connected with $S^{n}$ via the channels κ and μ, resp., and M is distributed on a set M with $\frac{1}{n}\log\left|M\right|>R-\delta$ and with a suitable (deterministic) decoder $d:Y^{n}\to M$,*

(35a) $$\begin{array}{cc} \;Pr\;\left[d(Y^{n})\neq M\right]<\epsilon & \;(\mathrm{reliability}), \end{array}$$

(35b) $$\begin{array}{cc} H(M|Z^{n})>\log\left|M\right|-\epsilon & \;(\mathrm{strong}\mathrm{secrecy}). \end{array}$$

Equation (35a) ensures that the Bob’s probability of error is arbitrarily small while (35b) ensures that Eve has negligible information about the message.

The secrecy capacity of the wiretap channel admits the following characterization.

**Theorem** **5** ([65], Corollary 2)**.** *The secrecy capacity $C_{S}\left(S;Y|Z\right)$ of the wiretap channel is*

(36) $$\begin{array}{c} C_{S}\left(S;Y|Z\right)=\max\left[I(U;Y)-I(U;Z)\right] \end{array}$$

*for random variables (U,S,Y,Z) such that U−S−YZ is a Markov chain and $P_{Y|S}=\kappa$, $P_{Z|S}=\mu$. The auxiliary variable U may be assumed to have a range of size at most |S|.*

$C_{S}$ depends on ξ only through its marginals κ and μ [65]. When the distribution of the input to ξ is fixed, $C_{S}$ depends only on the marginal distributions of the pairs (S,Y) and (S,Z). Hence, we can analyze if secure communication is possible or not by restricting our attention to $\Delta_{P}$ (see Definition 1). Proposition 9 shows that one can interpret the quantity $C_{S}$ as quantifying a deviation from the less noisy order.

**Proposition** **9** ([65], Corollary 3)**.**

(37) $$\begin{array}{c} \mu {⊒}^{\ln}\kappa \Leftrightarrow C_{S}\left(S;Y|Z\right)=0. \end{array}$$

The setting originally considered by Wyner [64] is a special case of the wiretap channel model where Eve’s channel is *physically degraded* from the main channel in the sense that ξ=κ×λ for some λ∈M(Y;Z). We call this the *degraded wiretap channel* model. The binary erasure wiretap channel in Example 6 is an instance of this model where κ is a noiseless channel and μ=BEC(ϵ). The coding scheme in this example is apparently not that useful since the transmission rate goes to zero as *n* goes to infinity, albeit more slowly than does $I(M;Z^{n})$. Nevertheless, the example suggests that when Alice is allowed to use a *stochastic* encoder, she can map a given message to a bin of codewords, and then select one of them at random to “confuse” Eve and achieve some secrecy guarantee. This intuition is brought to bear by Wyner, who showed that it is possible to transmit at a rate bounded away from zero and still achieve some secrecy guarantee by using a random binning scheme.

The secrecy capacity of the degraded wiretap channel is

(38) $$\begin{array}{c} C_{S}^{w}\left(S;Y|Z\right)=\max_{P_{S}}\left[I(S;Y)-I(S;Z)\right]=\max_{P_{S}}I\left(S;Y|Z\right), \end{array}$$

where the second equality follows from the fact S−Y−Z is a Markov chain by assumption. Note that $C_{S}\left(S;Y|Z\right)\geq C_{S}^{w}\left(S;Y|Z\right)$ since U=S is a valid choice in (36). Also note that if Eve obtains the same information as Bob; i.e., if Z=Y, then $C_{S}^{w}=C_{S}=0$. This is consistent with our analysis of Shannon’s model and the general idea that it is *impossible* to realize unconditional security “from scratch”, i.e., if only public channels are available.

For jointly distributed random variables (S,Y,Z)∼P, I(S;Y|Z) is a concave function of $P_{S}$ for fixed $P_{YZ|S}$ (see Lemma 3.3 in [13]). Thus, the optimization problem in (38) is a convex program. We can also relate $C_{S}^{w}$ to the main channel capacity $C_{\kappa }:=\max_{P_{S}}I\left(S;Y\right)$ and to Eve’s channel capacity $C_{\mu }:=\max_{P_{S}}I\left(S;Z\right)$ as follows:

$$\begin{array}{c} C_{S}^{w}\left(S;Y|Z\right)=\max_{P_{S}}\left[I(S;Y)-I(S;Z)\right]\geq \max_{P_{S}}I\left(S;Y\right)-\max_{P_{S}}I\left(S;Z\right)=C_{\kappa }-C_{\mu }. \end{array}$$

The secrecy capacity of the degraded wiretap channel is hence at least as large as the difference between the main channel capacity and Eve’s channel capacity. Note that if μ is physically degraded from κ, then $\kappa {⊒}_{S}^{\mathrm{odeg}}\mu$, but not conversely. However, since $C_{S}$ and $C_{S}^{w}$ depend on ξ only through its marginals κ and μ, there is no real difference between output-degraded channels and physically degraded channels from the point of view of secure communication.

For the models discussed so far, a necessary condition for Alice and Bob to be able to communicate in secrecy is that they have an explicit *physical advantage* over Eve. In Shannon’s model, for instance, Alice and Bob need to share a secret key in advance, while in Wyner’s model, the main channel must be less noisier than Eve’s. An obvious weakness of these models is that in a practical application, it may not often be possible to guarantee such an advantage. A key question is whether Alice and Bob can exchange messages in secrecy when they do *not* have a physical advantage to start with. Consider the following example:

**Example** **7** (Binary broadcast channel with independent BSCs, Lemma 1 in [85])**.** *Let ξ=κ×μ where κ=BSC(ϵ) and μ=BSC(δ) and $\epsilon \leq \frac{1}{2}$, $\delta \leq \frac{1}{2}$. The secrecy capacity of ξ is*

(39) $$\begin{array}{c} C_{S}\left(S;Y|Z\right)=\left\{\begin{array}{cc} h(\delta )-h(\epsilon ), & if\;\delta >\epsilon \\ 0, & \mathrm{otherwise} \end{array}\right. \end{array}$$

*$C_{S}$ vanishes whenever Bob’s channel is noisier than Eve’s in the sense that δ≤ϵ. Here, h(·) is the binary entropy function.*

Consider now a variation in the scenario in Example 7, where Bob can also send messages to Alice over an insecure public channel. We are interested in whether secrecy guarantees are possible in the range $0<\delta \leq \epsilon <\frac{1}{2}$ for this augmented scenario. The following example, due to Maurer [85], shows an ingenious trick to achieve this.

**Example** **8** (Public feedback from Bob to Alice increases secrecy capacity [85])**.** *Alice inputs a random bit S to the “real” channel ξ where $S∼\mathrm{Bernoulli}\;\left(\frac{1}{2}\right)$. Let $E∼\mathrm{Bernoulli}\;\left(\epsilon \right)$ and $D∼\mathrm{Bernoulli}\;\left(\delta \right)$ be, resp., the independent error bits of the main channel and Eve’s channel. Bob observes Y=S⊕E and Eve observes Z=S⊕D. We assume that the main channel is noisier than Eve’s in the sense that δ≤ϵ.*

*To send a message bit C, Bob computes W=C⊕Y=C⊕S⊕E and sends it over the public channel. Since Alice knows S, she computes W⊕S=C⊕E. Eve, on the other hand, only knows Z, and she computes W⊕Z=C⊕E⊕D. In effect, this procedure simulates a “conceptual” broadcast channel from Bob to Alice and Eve, where the conceptual main channel (to Alice) is equivalent to the real main channel and Eve’s conceptual channel is a composition of the real main channel and Eve’s real channel. This corresponds exactly to Wyner’s degraded wiretap channel scenario, where Eve’s conceptual channel is physically degraded from the main channel, thus allowing for some positive secrecy rate. Maurer showed that a suitably modified notion of secrecy capacity (called the* secrecy capacity with public discussion*) for this augmented scenario is equal to h(ϵ+δ−2ϵδ)−h(ϵ), which is strictly positive unless $\epsilon =\frac{1}{2}$, δ=0 or δ=1, (see Proposition 1 in* [85]*).*

Example 8 highlights the important fact that noiseless feedback can increase the secrecy capacity. This is true even when the feedback is known to Eve and she has a physical advantage over Bob. Crucially, the latter finding suggests that the necessity of the condition that Bob has a physical advantage over Eve to achieve a positive secrecy capacity in Example 7 stems from a restriction imposed by rate-limited one-way communication. These observations motivate the study of more general models of secret key agreement using two-way or interactive public communication.

**The source model for secret key agreement using public discussion.** Maurer introduced the * source model * for secret key agreement [12,85]. In this model, Alice, Bob, and Eve observe *n* i.i.d. copies of random variables *S*, *Y*, and *Z*, respectively, where (S,Y,Z) follows a joint distribution known to all parties, referred to as the *source*. Alice and Bob aim to agree on a common secret key by communicating interactively over a public channel that is observable by Eve.

The *two-way* public communication protocol proceeds in rounds, with Alice and Bob alternately exchanging messages. Alice sends messages in the odd-numbered rounds, and Bob sends messages in the even-numbered rounds. Each message is a function of the sender’s observation and all previously exchanged messages. At the conclusion of the protocol, Alice (resp. Bob) computes a key *K* (resp. $K^{\prime }$) as a function of $S^{n}$ (resp. $Y^{n}$) and *C*, the set of all exchanged messages.

**Definition** **19** ([85])**.** *The* two-way secret key rate *for the source model, denoted as $S_{↔}\;\left(S;Y\;\left|Z\right.\right)$, is the maximum rate R such that for every ϵ>0 and sufficiently large n, there exists a two-way public communication protocol that outputs keys K and $K^{\prime }$ (ranging over some common set K) satisfying*

(40a) $$\begin{array}{cc} \;Pr\;\left[K=K^{\prime }\right]\geq 1-\epsilon & \;(\mathrm{reliability}), \end{array}$$

(40b) $$\begin{array}{cc} \frac{1}{n}I\left(K;C,Z^{n}\right)\leq \epsilon & \;(\mathrm{weak}\mathrm{secrecy}), \end{array}$$

(40c) $$\begin{array}{cc} \frac{1}{n}H\left(K\right)>\frac{1}{n}\log\left|K\right|-\epsilon & \;(\mathrm{uniformity}), \end{array}$$

*and achieving $\frac{1}{n}H\left(K\right)\geq R-\epsilon$, where C is the amount of public communication consumed in the protocol.*

Equations (40a) and (40c) ensure, resp., that the keys are equal to each other with high probability and that they are almost uniformly distributed. Equation (40b) ensures that the *rate* at which Eve learns information about the keys is negligibly small. A still stronger definition requires that Eve’s *total* information about the key is negligibly small, i.e.,

(41) $$\begin{array}{c} I\left(K;C,Z^{n}\right)\leq \epsilon ;\;\;\;\;\;\;\;\;\;\;\left(\mathrm{strong}\mathrm{secrecy}\right). \end{array}$$

Both these definitions give the same secret key rate [98]. Moreover, this rate is achievable without using private randomness at either Alice’s or Bob’s end. This is unlike the wiretap channel model, where coding schemes for the strong secrecy and the weak secrecy condition are very different [13] and randomness in the encoding process plays a crucial role in enabling secure communication.

Note that Definition 19 of the two-way rate says nothing about the amount of public communication (i.e., the number of rounds) required to agree on a secret key, which can be arbitrarily large. However, models imposing some restriction on the possible communication are also of interest. We say that the protocol is *one-way* if Alice is allowed to send only one message and Bob none. The corresponding key rate is called the *one-way secret key rate* $S_{\to }\;\left(S;Y\;\left|Z\right.\right)$. The one-way key rate is a lower bound on the two-way key rate. $S_{\to }$ admits the following characterization.

**Theorem** **6** ([66], Theorem 1)**.** *The* one-way secret key rate *$S_{\to }\;\left(S;Y\;\left|Z\right.\right)$ for the source model is the solution of the following optimization problem:*

(42) $$\begin{array}{cc} S_{\to }\;\left(S;Y\;\left|Z\right.\right)=\max_{P_{UV|SYZ}:V-U-S-YZ} & I(U;Y|V)-I(U;Z|V). \end{array}$$

*In this optimization problem, it suffices to restrict the range of the random variables U and V to sizes ${\left|S\right|}^{2}$ and |S|, respectively.*

The bounds on the cardinalities imply that the optimization domain is a set of stochastic matrices of finite size, which makes it possible to turn this theorem into an algorithm to compute $S_{\to }$.

The following trivial bounds on the two-way rate are known [85]:

**Proposition** **10.**
$$\begin{array}{c} \max\left\{I\left(S;Y\right)-I\left(S;Z\right),I\left(Y;S\right)-I\left(Y;Z\right)\right\}\leq S_{↔}\;\left(S;Y\;\left|Z\right.\right)\leq \min\left\{I\left(S;Y\right),I\left(S;Y|Z\right)\right\}. \end{array}$$

For some sources, the lower bound in Proposition 10 can be negative (see [99] for an operational interpretation of the lower bound when such is the case). If neither I(S;Y)>I(S;Z) nor I(Y;S)>I(Y;Z) holds, then Alice and Bob can exploit the authenticity of the public channel to “distill” observations for which Alice and Bob have an advantage over Eve.

Maurer [85] and Maurer and Wolf [100] considered a scenario where a satellite broadcasts random bits at a low signal power and earthlings Alice, Bob, and Eve receive these bits over independent binary channels. Secret key agreement is *always* possible in this scenario unless Eve’s channel is noiseless or either Alice or Bob receives no information at all about these bits. The following example describes an advantage distillation strategy called the “repeat-code protocol” for this scenario.

**Example** **9** (The “satellite” source with independent BSCs [85,100])**.** *Let R∼$\mathrm{Bernoulli}\;\;\left(\frac{1}{2}\right)$. We pass R through three independent binary symmetric channels with parameters α, β, and ϵ, resp., to obtain S, Y, and Z. We assume that $0\leq \alpha ,\beta <\frac{1}{2}$, and 0<ϵ<min{α,β}. Thus, Eve has an initial advantage over Alice and Bob in the sense that I(S;Z)>I(S;Y) and I(Y;Z)>I(Y;S).*

*Given n realizations of the source, Alice and Bob exploit the authenticity of the public channel to reverse Eve’s advantage as follows: Alice generates a bit C∼$\mathrm{Bernoulli}\;\;\left(\frac{1}{2}\right)$ and sends $S^{n}⊕C^{n}$ over the public channel, where *⊕* denotes a bit-wise* Xor *operation and $C^{n}$ is a vector consisting of n repetitions of the bit C. Bob computes $\left(S^{n}⊕C^{n}\right)⊕Y^{n}$ and publicly “accepts” if and only if his output is equal to either (0,0,…,0) or (1,1,…,1), when Alice retains C; or else, Alice discards C. In other words, Alice and Bob make use of a code comprising two n-bit codewords (0,0,…,0) and (1,1,…,1) and retain a bit only if their observations are either highly correlated or highly anti-correlated. Eve computes $\left(S^{n}⊕C^{n}\right)⊕Z^{n}$ and her optimal guess for C is *0* if at least half of the bits in her string is *0*, and *1* otherwise. As n goes to infinity, Bob’s average error probability when guessing the bit C sent by Alice decreases asymptotically faster than Eve’s and that the secret key rate is strictly positive in this scenario. This protocol can be used over multiple rounds to further reduce Eve’s information.*

The design of practical secret key agreement protocols turns out to be a simpler problem than the construction of wiretap channel codes [13]. A wiretap code needs to *simultaneously* guarantee reliable communication of a message to Bob (35a) and secrecy against Eve (35b). On the other hand, keys are random strings that are not meant to convey any information by themselves and do not need to be known in advance. Alice and Bob can freely shuffle, combine, or discard their observations. This allows for the design of *sequential* key distillation strategies that handle the reliability constraint (40a) and secrecy constraint (40b) *independently*. Since one can always post-process weakly secret keys, strong secrecy comes “for free,” i.e., a rate achievable under the weak secrecy condition (40b) is also achievable under the strong secrecy condition (41) (see Theorem 1 in [98]).

A typical key agreement protocol operates in sequential phases [13]: First, Alice, Bob, and Eve observe *n* realizations of a source. Second, if neither Alice nor Bob has an initial advantage over Eve, they use an *advantage distillation* strategy to reverse Eve’s advantage. Third, Alice and Bob exchange messages over the public channel and apply error correction techniques to process their observations and agree on a common bit string. This phase is called *information reconciliation*. Since the error correction information is public, the common bits are only partially secret from Eve. Fourth, Alice and Bob use a suitable hash function to distill a (shorter) highly secret string about which Eve has virtually no information. This phase is called *privacy amplification by public discussion* [101]. Finally, they use the key as an OTP for secure encryption.

**The channel model for secret key agreement using public discussion.** A *channel model* for secret key agreement generalizes the source model [66,85]. The model involves a channel ξ∈M(S;Y×Z). Alice selects the inputs to ξ, while Bob and Eve observe, resp., the corresponding *Y*-outputs and the *Z*-outputs of ξ. Alice and Bob also have access to a public channel. The definitions of the two-way and one-way secret key rates are similar to those for the source model (see Section 17.3 in [27]). Given a channel model, Alice can emulate the associated source model by choosing i.i.d. copies of a random variable *S* as inputs to ξ. The corresponding channel outputs are i.i.d. copies of *Y* and *Z*. Hence, any key rate achieved by a source model with generic variables (S,Y,Z) subject to $P_{YZ|S}=\xi$ is also achieved by the associated channel model [66].

The wiretap channel model may be regarded as a channel model where no public communication is allowed. Clearly, the secret key rate for the channel model defined by ξ is at least as large as the secrecy capacity $C_{S}$ of the associated wiretap channel defined by the components κ and μ of ξ (see Theorem 5). Ahlswede and Csiszár [66] showed that the one-way secret key rate for the channel model is equal to the secrecy capacity of the associated wiretap channel model. Thus, the one-way rate depends on ξ only through κ and μ. Note, however, that the same is not true for the two-way rate [27].

The secret key rate for the channel model is sometimes called the secrecy capacity with public discussion [85] (e.g., see Example 8). This denomination is slightly misleading because the former characterizes a secret key rate, not a secure communication rate [13].

In the sequel, we shall concern ourselves primarily with the source model.

### 5.3. Known Bounds on the Two-Way Secret Key Rate

#### 5.3.1. Lower Bounds

The best-known lower bound on $S_{↔}$ uses two-way public communication [67,80]. Given random variables $U_{1},U_{2},\cdots ,U_{k}$ satisfying the Markov chain conditions

(43) $$\begin{array}{cc} \; & U_{i}-SU_{1:i-1}-YZ,\;\mathrm{for}\;\mathrm{odd}\;i \end{array}$$

(44) $$\begin{array}{cc} \; & U_{i}-YU_{1:i-1}-SZ,\;\mathrm{for}\;\mathrm{even}\;i \end{array}$$

and for any integer ζ such that 1≤ζ≤k, we have $S_{↔}\;\left(S;Y\;\left|Z\right.\right)\geq L\left(S;Y|Z\right)$ where

(45) $$\begin{array}{cc} & L\left(S;Y|Z\right)=\sum_{\begin{array}{c} i\geq \zeta \\ \;\mathrm{odd}\;i \end{array}}I\left(U_{i};Y|U_{1:i-1}\right)-I\left(U_{i};Z|U_{1:i-1}\right)\;+\;\sum_{\begin{array}{c} i\geq \zeta \\ \;\mathrm{even}\;i \end{array}}\;I\left(U_{i};S|U_{1:i-1}\right)\;-\;I\left(U_{i};Z|U_{1:i-1}\right), \end{array}$$

and the cardinality bounds on $U_{1},U_{2},\ldots ,U_{k}$ satisfy

(46) $$\begin{array}{c} |U_{i}|\leq \left\{\begin{array}{cc} \left|S\right|\prod_{l=1}^{i-1}\left|U_{l}\right| & \mathrm{for}\;i\;\mathrm{odd},\\ \left|Y\right|\prod_{l=1}^{i-1}\left|U_{l}\right| & \mathrm{for}\;i\;\mathrm{even}. \end{array}\right. \end{array}$$

The bound (45) is difficult to evaluate but is quite intuitive: depending on whether *i* is odd or even, the individual terms can be understood from the form of the one-way secret key rate in Theorem 6 when either Alice or Bob sends a public message.

#### 5.3.2. Upper Bounds

As noted in Proposition 10, a trivial upper bound on $S_{↔}\;\left(S;Y\;\left|Z\right.\right)$ is min{I(S;Y),I(S;Y|Z)} [85].

The two-way rate equals the conditional mutual information when Eve helps Alice and Bob by announcing her variable, i.e., $S_{↔}\;\left(SZ;YZ\;\left|Z\right.\right)=I\left(S;Y|Z\right)$. This ascribes an operational meaning to I(S;Y|Z) as the key rate obtained when Alice and Bob have an explicit advantage over Eve.

If Eve sends *Z* through a channel $P_{Z^{\prime }|Z}$, then the key rate cannot decrease. Thus, we have $S_{↔}\;\left(S;Y\;\left|Z\right.\right)\leq S_{↔}\;\left(S;Y\;\left|Z^{\prime }\right.\right)\leq I\left(S;Y|Z^{\prime }\right)$ for any $P_{Z^{\prime }|Z}$ [12]. This observation motivates an improved bound by way of the *intrinsic information*, $I_{↓}$:

(47) $$\begin{array}{c} S_{↔}\;\left(S;Y\;\left|Z\right.\right)\leq I\left(S;Y\;\;↓\;Z\right):=\min_{P_{Z^{\prime }|Z:SY-Z-Z^{\prime }}}I\left(S;Y|Z^{\prime }\right). \end{array}$$

where $Z^{\prime }$ may be assumed to have a range of size at most |Z| [102]. Unlike the UI, which depends only on the marginal distributions of the pairs (S,Y) and (S,Z), $I_{↓}$ depends on the full joint distribution, and also does not satisfy the consistency condition (7). Proposition 11 shows that $I_{↓}$ is never lower than the UI.

**Proposition** **11.** *UI(S;Y\Z)≤I(S;Y↓Z).*

See Appendix B for a proof.

Renner and Wolf [84] noted that the intrinsic information exhibits a property called “locking”; i.e., it can drop by an arbitrarily large amount on giving away a bit of information to Eve. In contrast, the two-way rate satisfies

(48) $$\begin{array}{c} S_{↔}\;\left(S;Y\;\left|ZU\right.\right)\geq S_{↔}\;\left(S;Y\;\left|Z\right.\right)-H\left(U\right) \end{array}$$

for jointly distributed random variables (S,Y,Z,U) (see Theorem 3 in [84]), and the conditional mutual information satisfies an analogous property:

(49) $$\begin{array}{c} I(S;Y|ZU)\geq I(S;Y|Z)-H(U). \end{array}$$

Renner and Wolf [84] proposed an improved upper bound called the *reduced intrinsic information* $I_{↓↓}$, which does not exhibit locking:

(50) $$\begin{array}{cc} I(S;Y\;\;↓↓\;Z) & :=\underset{P_{U|SYZ}}{\inf}I\left(S;Y\;\;↓\;ZU\right)+H\left(U\right)\\ & \geq \underset{P_{U|SYZ}}{\inf}S_{↔}\;\left(S;Y\;\left|ZU\right.\right)+H\left(U\right)=S_{↔}\;\left(S;Y\;\left|Z\right.\right). \end{array}$$

Choosing *U* to be a constant, one immediately obtains I(S;Y↓↓Z)≤I(S;Y↓Z). I(S;Y↓↓Z) does not lock since

$$\begin{array}{cc} I\left(S;Y\;\;↓↓\;Z\right)=\underset{P_{V|SYZ}}{\inf}I\left(S;Y\;\;↓\;ZV\right)+H\left(V\right) & \leq \underset{P_{U^{\prime }|SY\left(Z,U\right)}}{\inf}I\left(S;Y\;\;↓\;ZUU^{\prime }\right)+H\left(UU^{\prime }\right)\\ & \leq I(S;Y\;\;↓↓\;ZU)+H(U), \end{array}$$

where the inequality in the second step follows from restricting the infimum to random variables $V=UU^{\prime }$.

The tightest known upper bound on the two-way rate is [67]

(51) $$B_{2}\left(S;Y|Z\right):=\underset{p_{Z^{\prime }|SYZ}}{\inf}I\left(S;Y|Z^{\prime }\right)+S_{\to }\;\left(SY;Z^{\prime }\;\left|Z\right.\right).$$

Unfortunately, $B_{2}$ cannot be computed explicitly, as no bound on the size of $Z^{\prime }$ is known.

A slightly weaker but computable upper bound is given by the *minimum intrinsic information* [67].

(52) $$\begin{array}{c} B_{1}\left(S;Y|Z\right):=\min_{P_{Z^{\prime }|SYZ}}I\left(S;Y|Z^{\prime }\right)+I\left(SY;Z^{\prime }|Z\right), \end{array}$$

where $|Z^{\prime }\left|\leq |S||Y||Z\right|$.

Summarizing, we have the following chain of bounds on the two-way rate.

(53) $$\begin{array}{cc} \;\;C_{S}\left(S;Y|Z\right)\leq S_{\to }\;\left(S;Y\;\left|Z\right.\right)\leq L\left(S;Y|Z\right) & \leq S_{↔}\;\left(S;Y\;\left|Z\right.\right)\\ & \leq B_{2}\left(S;Y|Z\right)\leq B_{1}\left(S;Y|Z\right)\\ \; & \leq I(S;Y\;\;↓↓\;Z)\leq I(S;Y\;\;↓\;Z)\leq I(S;Y|Z). \end{array}$$

### 5.4. Properties of the UI

In this section, we show that the function UI shares some fundamental properties of the secret key rate.

We first recall the trivial bounds on the UI [6]:

(54) $$\begin{array}{c} I(S;Y)-I(S;Z)\leq UI(S;Y\backslash Z)\leq \min\{I(S;Y),I(S;Y|Z)\}. \end{array}$$

These bounds match the trivial bounds on the two-way secret key rate in Proposition 10 (note that $S_{↔}\;\left(S;Y\;\left|Z\right.\right)$ is symmetric under permutations of *S* and *Y*, while UI(S;Y\Z) is not). In the adversarial setting in Example 4, if either Eve has less information about *S* than Bob or, by symmetry, less information about *Y* than Alice, then Alice and Bob can exploit this difference to extract a secret key.

Property P.1 states that the UI does not exhibit locking.

P.1 (*UI does not lock*). For jointly distributed random variables (S,Y,Z,U),
  (55) $$\begin{array}{c} UI(S;Y\backslash ZU)\geq UI(S;Y\backslash Z)-H(U). \end{array}$$

This property is useful as it ensures that the unique information that *Y* has about *S* with respect to an adversary *Z* cannot “unlock”, i.e., drop by an arbitrarily large amount on giving away some information to *Z*.

Property P.1 and Proposition 11 together imply that UI(S;Y\Z)≤I(S;Y↓↓Z), a fact that will be generalized later in Theorem 9.

Property P.2 states that UI can never increase under local operations of Alice and Bob. The counterpart of this property for the secret key rate is Lemma 4 in [12]. On a related note, in Section 6.3, we discuss a construction that enforces monotonicity under local operations for an arbitrary information measure.

P.2 (*Monotonicity under local operations (LOs) of Alice and Bob*). For all $\left(S,S^{\prime },Y,Z\right)$ such that YZ–*S*–$S^{\prime }$ is a Markov chain, $UI\left(S;Y\backslash Z\right)\geq UI\left(S^{\prime };Y\backslash Z\right)$. Likewise, for all $\left(S,Y,Y^{\prime },Z\right)$ such that SZ–*Y*–$Y^{\prime }$ is a Markov chain, $UI\left(S;Y\backslash Z\right)\geq UI\left(S;Y^{\prime }\backslash Z\right)$.

Suppose Alice publicly announces the value of a random variable. Then, Property P.3 states that UI can never increase.

P.3 (*Monotonicity under public communication (PC) by Alice*). For all (S,Y,Z) and functions *f* over the support of *S*, UI((S,f(S));(Y,f(S))\(Z,f(S)))≤UI(S;Y\Z).

The basic unit of secrecy is the “secret bit” Φ. This is any distribution defined on the sets {0,1}×{0,1}×Z such that

(56) $$\begin{array}{c} \Phi \left(s,y,z\right):=\frac{1}{2}\delta_{s,y}\times Q_{Z}\left(z\right), \end{array}$$

where $Q_{Z}$ is an arbitrary distribution.

For the secret bit, UI satisfies an intuitive normalization property:

P.4 (*Normalization*). $UI_{\Phi }\left(S;Y\backslash Z\right)=UI_{\Phi }\left(Y;S\backslash Z\right)=1$.

Given many independent copies of (S,Y,Z)∼*P*, the goal of a secret key agreement protocol is to distill as many copies of Φ as possible. The following two properties, additivity and asymptotic continuity, are important since we are concerned with the asymptotic rate of secret key distillation.

P.5 (*Additivity on tensor products*). Let random variables $\left(S,S^{\prime },Y,Y^{\prime },Z,Z^{\prime }\right)$ be such that (S,Y,Z) is independent of $\left(S^{\prime },Y^{\prime },Z^{\prime }\right)$. Then, $UI\left(SS^{\prime };YY^{\prime }\backslash ZZ^{\prime }\right)=UI\left(S;Y\backslash Z\right)+UI\left(S^{\prime };Y^{\prime }\backslash Z^{\prime }\right)$.

Property P.5 is shown in Lemma 19 of [6].

Asymptotic continuity is a stronger form of continuity that takes into account convergence in relation to the dimension of the underlying state space [11,18,103,104,105]. Specifically, a function *f* is said to be asymptotically continuous if

$$|f\left(P\right)-f\left(P^{\prime }\right)|\leq C\epsilon \log|S|+\zeta \left(\epsilon \right)$$

for all joint distributions $P,P^{\prime }\in P_{S}$, where *C* is a constant, $\epsilon =\frac{1}{2}{∥P-P^{\prime }∥}_{1}$, and $\zeta :\left[0,1\right]\to R_{+}$ is a continuous function that converges to zero as ϵ→0 [11].

As an example, entropy is asymptotically continuous (see, e.g., Lemma 2.7 in [27]): for any $P,P^{\prime }\in P_{S}$, if $\frac{1}{2}{∥P-P^{\prime }∥}_{1}\leq \epsilon$, then

$$|H_{P}\left(S\right)-H_{P^{\prime }}\left(S\right)|\leq \epsilon \log|S|+h\left(\epsilon \right),$$

where h(·) is the binary entropy function. Likewise, the conditional mutual information satisfies asymptotic continuity in the following sense [84,106]: for any $P,P^{\prime }\in P_{S\times Y\times Z}$, if $\frac{1}{2}{∥P-P^{\prime }∥}_{1}\leq \epsilon$, then

$$|I_{P}\left(S;Y|Z\right)-I_{P^{\prime }}\left(S;Y|Z\right)|\leq \epsilon \log\min\{|S|,|Y|\}+2h\left(\epsilon \right).$$

Note that the right-hand side of the above inequality does not depend explicitly on the cardinality of *Z*.

The function UI is as asymptotically continuous:

P.6 (*Asymptotic continuity*). For any $P,P^{\prime }\in P_{S\times Y\times Z}$ and ϵ∈[0,1], if $∥P-P^{\prime }{∥}_{1}=\epsilon$, then ${UI}_{P^{\prime }}\left(S;Y\backslash Z\right)-{UI}_{P}\left(S;Y\backslash Z\right)\leq \zeta \left(\epsilon \right)+\frac{5}{2}\epsilon \log\min\left\{|S|,|Y|\right\}$ for some bounded, continuous function $\zeta :\left[0,1\right]\to R_{+}$ such that ζ(0)=0.

The function UI satisfies a triangle inequality:

P.7 (*Triangle inequality*). For any $\left(S,Y,Z,Z^{\prime }\right)$,
  $$UI\left(S;Y\backslash Z\right)\leq UI\left(S;Y\backslash Z^{\prime }\right)+UI\left(S;Z^{\prime }\backslash Z\right).$$

An intuitive understanding of Property (P.7) can be gained by iterating the fundamental idea of information decomposition as follows: In the presence of a fourth variable $Z^{\prime }$, we aim to decompose u:=UI(S;Y\Z) into two components—a part $u_{1}$, which is also known to $Z^{\prime }$, and the remainder $u_{2}=u-u_{1}$, which $Z^{\prime }$ does not know. Clearly, $u_{1}$ should be upper-bounded by $UI(S;Z^{\prime }\backslash Z)$, as $Z^{\prime }$ alone knows what $Z^{\prime }$ and *Y* share. Moreover, $u_{2}\leq UI\left(S;Y\backslash Z^{\prime }\right)$, since what neither *Z* nor $Z^{\prime }$ knows is less than what $Z^{\prime }$ does not know. Together, these observations provide a heuristic argument for why the triangle inequality should hold.

Property P.7 relies on the following monotonicity property: UI can only increase under local operations by Eve.

P.8 (*Monotonicity under local operations of Eve*). For all $\left(S,Y,Z,Z^{\prime }\right)$ such that SY–*Z*–$Z^{\prime }$ is a Markov chain, $UI\left(S;Y\backslash Z\right)\leq UI\left(S;Y\backslash Z^{\prime }\right)$.

Using Property P.7 and Property P.2, we conclude:

**Corollary** **1.** *For any $\left(S,Y,Z,Z^{\prime }\right)$, $UI\left(S;Y\backslash Z\right)\leq UI\left(S;Y\backslash Z^{\prime }\right)+UI\left(SY;Z^{\prime }\backslash Z\right).$*

We can interpret Corollary 1 like inequality (33): Given $(S,Y,Z,Z^{\prime })∼P$, if the induced channel $P_{Z|SY}$ dominates the channel $P_{Z^{\prime }|SY}$ in the Blackwell sense, then the second term $UI(SY;Z^{\prime }\backslash Z)$ vanishes (see Lemma 1). One can interpret $UI(SY;Z^{\prime }\backslash Z)$ as quantifying a deviation from the Blackwell order when we replace $P_{Z|SY}$ with $P_{Z^{\prime }|SY}$.

### 5.5. UI-Based Bounds on Secret Key Rates

General properties of upper bounds on secret key rates have been studied within the framework of secrecy or protocol monotones—non-negative real-valued functionals of joint distributions that remain non-increasing throughout the execution of a protocol (see, e.g., [14,67,88,89,107]). For example, the *intrinsic information* in (47) is a protocol monotone [84]. We defer a more general discussion on protocol monotones in the context of *resource theories* to Section 6.

The following theorem gives sufficient conditions for a function to be an upper bound for the secret key rate.

**Theorem** **7** (Theorem 3.1 in [107], Lemma 2.10 in [88])**.** *Let M be a non-negative real-valued function of the joint distribution of the triple (S,Y,Z) that satisfies Properties P.2–P.6. Then, M is an upper bound for the one-way secret key rate.*

*If, in addition, M does not increase under public communication by Bob (Property P.3, with f(S) replaced by g(Y) for some function g over the support of Y), then M is an upper bound for the two-way secret key rate.*

Like the UI, $S_{\to }$ depends only on the marginal distributions of the pairs (S,Y) and (S,Z) [66]. Since UI satisfies Properties P.2–P.6, the following result is immediate from Theorem 7:

**Theorem** **8.** *UI(S;Y\Z) is an upper bound for the one-way secret key rate $S_{\to }\;\left(S;Y\;\left|Z\right.\right)$.*

Corollary 1 implies the following result.

**Proposition** **12.** *
$UI\left(S;Y\backslash Z\right)\leq B_{1}\left(S;Y|Z\right).$
*

From Theorem 8 and Proposition 12, we have the following chain of inequalities relating the bounds on the two-way rate.

**Theorem** **9.**
(57) $$\begin{array}{cc} \;\;C_{S}\left(S;Y|Z\right)\leq S_{\to }\;\left(S;Y\;\left|Z\right.\right) & \leq UI\left(S;Y\backslash Z\right)\leq B_{1}\left(S;Y|Z\right)\\ \; & \leq I(S;Y\;\;↓↓\;Z)\leq I(S;Y\;\;↓\;Z)\leq I(S;Y|Z). \end{array}$$

**Remark** **4.** *Given (S,Y,Z)∼P, let*

(58) $$\begin{array}{c} Q^{*}\in \underset{Q\in \Delta_{P(S,Y,Z)}}{arg min}I_{Q}\left(S;Y|Z\right). \end{array}$$

By definition, $I_{Q^{*}}\left(S;Y|Z\right)=UI\left(S;Y\backslash Z\right)$. Recall that the distribution $Q^{*}$ is a minimum synergy *distribution (see Equation *(16)). An immediate consequence of Theorem 9 is as follows: choosing $P=Q^{*}$, all known upper bounds on the two-way rate collapse to the UI and the conditional mutual information, respectively.

The following example [80,108] shows that there exists a distribution for which UI(S;Y\Z) is *not* lower than L(S;Y|Z), the best-known lower bound on the two-way rate (see (45)).

**Example 10** (Doubly symmetric binary erasure (DSBE) source)**.** *Consider the DSBE source with parameters (p,ϵ) in Example 3.*

*If ϵ=0, we have Z=SY and $S_{↔}\;\left(S;Y\;\left|Z\right.\right)=0$, while if ϵ=1, we have Z=e and $S_{↔}\;\left(S;Y\;\left|Z\right.\right)=I\left(S;Y\right)$.*

*For this source, the two-way rate vanishes if and only if (see Theorem 14 in *[12]*)*

(59) $$\begin{array}{c} \epsilon \leq \frac{1-p}{p}. \end{array}$$

*On the other hand, both the one-way secret key rate $S_{\to }\;\left(S;Y\;\left|Z\right.\right)$ and the best-known lower bound L(S;Y|Z) vanish if and only if (see Theorem 7 in *[80]*)*

(60) $$\begin{array}{c} \epsilon \leq 4p(1-p), \end{array}$$

*while both UI(S;Y\Z) and UI(Y;S\Z) vanish if and only if*

(61) $$\begin{array}{c} \epsilon \leq 2(1-p). \end{array}$$

**Figure 3:**
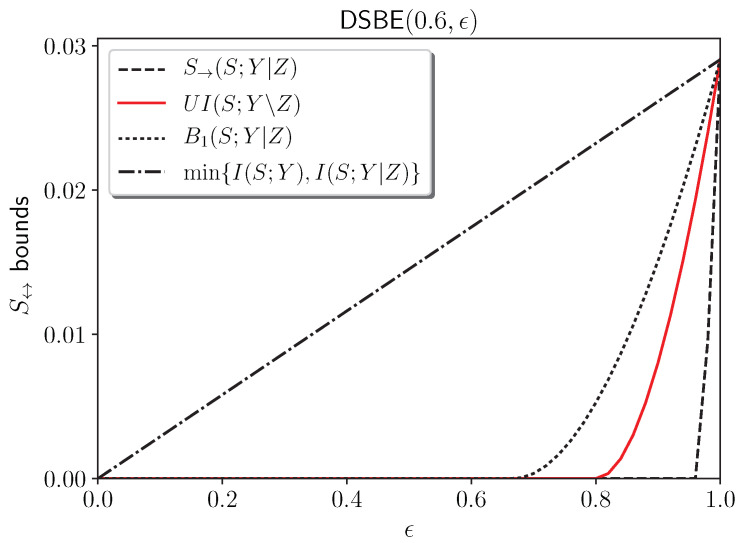
Bounds on the two-way secret key rate for a DSBE(0.6, ϵ) source.

*For $p>\frac{1}{2}$, we have $\frac{1-p}{p}<2\left(1-p\right)<4p\left(1-p\right)$. Figure 3 illustrates these bounds for a DSBE(0.6, ϵ) source.*

On the other hand, the following example shows that UI is *not* an upper bound on $S_{↔}$ (see also Example 12).

**Example** **11** (Satellite source with independent BECs [86])**.** *Let R∼$\mathrm{Bernoulli}\;\;\left(\frac{1}{2}\right)$. We pass R through three independent erasure channels with parameter ϵ to obtain S, Y, and Z. Thus, $P_{SYZR}\left(s,y,z,r\right)=P_{R}\left(r\right)P_{S|R}\left(s|r\right)P_{Y|R}\left(y|r\right)P_{Z|R}\left(z|r\right)$. Observe that $P_{SY}\left(a,b\right)=P_{SZ}\left(a,b\right)=P_{YZ}\left(a,b\right)$ for all a,b∈{0,1,e}. Therefore, all the UIs vanish. Gohari and Anantharam [86] showed that a secret key agreement protocol exists such that $S_{↔}\;\left(S;Y\;\left|Z\right.\right)=I\left(S;Y|Z\right)=\epsilon {\left(1-\epsilon \right)}^{2}$ is an achievable rate. $\epsilon {\left(1-\epsilon \right)}^{2}$ is strictly positive for ϵ∈(0,1).*

We make the following conjecture:

**Conjecture** **1.** $UI\left(S;Y\backslash Z\right)\leq S_{↔}\;\left(S;Y\;\left|Z\right.\right)$.

**Remark** **5** (Sandwich bound on $S_{↔}\;\left(S;Y\;\left|Z\right.\right)$)**.** *If Conjecture 1 is true, then*

(62) $$\begin{array}{c} UI\left(S;Y\backslash Z\right)=I_{Q^{*}}\left(S;Y|Z\right)\leq S_{↔}\;\left(S;Y\;\left|Z\right.\right)\leq I_{P}\left(S;Y|Z\right). \end{array}$$

*Equation *(62) implies that the set of all $Q^{*}$ as in (58)* is a set of distributions for which the UI equals the two-way rate.*

A related work [87] gives necessary conditions for when the two-way rate equals the conditional mutual information.

**Definition** **20.** *Define the following functions on $P_{S\times Y\times Z}$.*

$$\begin{array}{cc} B_{sUI}\left(S;Y|Z\right) & :\;=\underset{P_{Z^{\prime }|SYZ}}{\inf}UI\left(S;Y\backslash Z^{\prime }\right)+UI\left(SY;Z^{\prime }\backslash Z\right).\\ B_{gUI}\left(S;Y|Z\right) & :\;=\underset{P_{Z^{\prime }|SYZ}}{\inf}I\left(S;Y|Z^{\prime }\right)+UI\left(SY;Z^{\prime }\backslash Z\right). \end{array}$$

As the following proposition shows, $B_{gUI}\left(S;Y|Z\right)$ is a new upper bound on the two-way rate which is juxtaposed between the two best-known bounds $B_{2}$ and $B_{1}$.

**Proposition** **13.**
(63) $$\begin{array}{cc} B_{sUI}\left(S;Y|Z\right) & =UI\left(S;Y\backslash Z\right)\leq B_{gUI}\left(S;Y|Z\right) \end{array}$$

(64) $$\begin{array}{cc} B_{2}\left(S;Y|Z\right) & \leq B_{gUI}\left(S;Y|Z\right)\leq B_{1}\left(S;Y|Z\right) \end{array}$$

It remains to be seen if there exist scenarios where the $B_{gUI}$ bound is strictly better than the $B_{1}$ bound. This remains a scope for future study.

### 5.6. The Blackwell Property and Secret Key Agreement Against Active Adversaries

In the source model for secret key agreement, we assume that the public channel is authenticated; i.e., Eve is only a passive adversary. In practice, this is guaranteed by authentication schemes that require Alice and Bob to share a short secret key in advance [90]. However, if this assumption is no longer valid and Eve gains both read and write access to the public channel, Maurer and Wolf [109] established an all-or-nothing result: either the same secret key rate as in the authenticated channel case can be achieved, or no key can be established at all. Maurer introduced the following property of a joint distribution to characterize the impossibility of secret key agreement in the presence of an active adversary:

**Definition** **21.** *Given (S,Y,Z)∼P, we say that Y* is simulatable by *Z* with respect to *S and write ${sim}_{S}\left(Z\to Y\right)$ if there exists a random variable $Y^{\prime }$ such that the pairs (S,Y) and $\left(S,Y^{\prime }\right)$ are statistically indistinguishable, and $S-Z-Y^{\prime }$ is a Markov chain.*

It is immediately apparent that ${\mathrm{sim}}_{S}\left(Z\to Y\right)$ and $Z{⊒}_{S}^{\prime }Y$ in Definition 3 are equivalent. We now restate Maurer’s impossibility result in terms of the function UI. We write $S_{↔}^{*}$ to denote the secret key rate in the active adversary scenario.

**Theorem** **10** ([109], Theorem 11)**.** Let (S,Y,Z) be a triple of random variables such that $S_{↔}\;\left(S;Y\;\left|Z\right.\right)>0$. If either UI(S;Y\Z)=0 or UI(Y;S\Z)=0, then $S_{↔}^{*}\left(S;Y|Z\right)=0$, else $S_{↔}^{*}\left(S;Y|Z\right)=S_{↔}\;\left(S;Y\;\left|Z\right.\right)$.

**Remark** **6.** *Theorem 10 gives another operational interpretation of the vanishing UI; namely, if either S or Y possesses no unique information about each other with respect to Z, then Alice and Bob have no advantage in a secret key agreement task against an active Eve.*

Example 12 shows a distribution for which $S_{↔}\;\left(S;Y\;\left|Z\right.\right)>0$ but $S_{↔}^{*}\left(S;Y|Z\right)=0$.

**Example** **12** ([110], Example 4)**.** Consider the distribution $P_{SYZ}\left(0,0,0\right)=P_{SYZ}\left(0,0,1\right)=P_{SYZ}\left(0,1,0\right)=P_{SYZ}\left(1,0,0\right)=P_{SYZ}\left(1,1,1\right)=\frac{1}{5}$. This distribution has I(S;Y↓Z)=0.02. Gisin and Wolf [110] showed that a secret key agreement protocol exists such that $S_{↔}\;\left(S;Y\;\left|Z\right.\right)>0$. However, since the pairwise marginal distributions of (S,Y), (S,Z), and (Y,Z) are all identical, all the unique informations vanish. Thus, $S_{↔}^{*}\left(S;Y|Z\right)=0$.

For the passive key agreement scenario, Example 9 shows that two-round protocols can be strictly better than one-round protocols. In general, there exists no upper bound on the number of rounds required to agree on a secret key [111]. Orlitsky and Wigderson [112], however, gave a necessary and sufficient condition for the existence of a secret key: $S_{↔}>0$, if and only if $S_{↔}$ is positive with only *two rounds* of communication. Property P.3 shows that the UI can never increase in a one-round secret key agreement protocol where Alice sends a public message to Bob. An analysis of the behavior of the UI in two-round protocols, where, in addition, Bob feeds a message back to Alice, is reserved for future study.

## 6. Resource Theories of Secrecy

In this concluding section, we sketch the resource-theoretic underpinnings behind Theorem 8. Resource theories study a set of objects endowed with a preorder. Classical and quantum information theories can be viewed as examples of resource theories [113]. A resource-theoretic formulation of thermodynamics is implicit in Lieb and Yngvason’s axiomatic derivation of the second law of thermodynamics [114]. We refer the reader to [7,8,9,10,11] for detailed exposition on resource theories. We next study the problem of interconvertibility between a given pair of source and target distributions from a resource-theoretic perspective. This is similar in spirit to the work in [52] that briefly studied interconversions between the different partial information terms in (6) under local operations.

### 6.1. Theories of Resource Convertibility

Resource theories provide an abstract operational framework for studying what physical transformations between objects are possible under a certain class of constraints. The set of all possible operations on these objects can be divided into those that can be implemented in a cheap or simple way (called “free operations”), and those that entail a costly implementation. Given access to the set of free operations. the theory seeks to study the structure that is induced on the objects. We say that objects *A* and *B* are ordered as A→B, if *A* can be converted to *B* by free operations. An object is *free* if it can be generated from scratch using only free operations; all other objects are *resources*. The resource content of an object cannot increase under free operations.

For example, in the source model for secret key agreement (Section 5.2), the objects of interest are the set of all source distributions. The set of free operations is local operations and public communication (LOPC) by Alice and Bob. The free objects are the set of all distributions under which Alice and Bob’s observations are mutually independent; all other objects are *resources*. The basic resource unit is the secret bit Φ (see (56)). Resources are valuable in the sense that when combined with free operations, they can generate other resources or simulate non-free operations. For example, one can simulate a one-time pad (OTP) using LOPC and a secret bit (see Example 5).

Any non-negative, real-valued function *M* that respects the preorder in the sense that if A→B, then M(A)≥M(B) is called a *monotone*. *M* can be interpreted as an assignment of a value to each object in a way that is consistent with the preorder. If M(A)<M(B), then a conversion of *A* to *B* is not possible. This property is useful in practice for checking the infeasibility of a conversion.

When an *exact* conversion of *A* to *B* is not possible, we can instead ask for an *approximate* conversion at a many-copy level: convert *n* independent realizations of *A* to $B^{\prime }$ which is close to *m* independent realizations of the desired target *B*, i.e., $A^{⊗n}\to B^{\prime }≃B^{⊗m}$ under the free operations. The *rate* or *yield* of this conversion is $\gamma :=\frac{m}{n}$. The existence of a monotone that satisfies certain additivity and continuity properties allows us to obtain an upper bound on the rate of such conversions (Theorem 7).

The UI is a monotone that quantifies the resourcefulness or secrecy content of a source distribution when the set of free operations is local operations by Alice and Bob and one-way public communication by Alice. In particular, UI is non-increasing under this set of free operations. A consequence of this property is that the UI is an upper bound on the one-way secret key rate $S_{\to }$ (Theorem 8).

We now study two other “symmetric” monotones, the total correlation (TC) (see (65)) and the dual total correlation (DTC) (see (66)), which can be viewed as multipartite generalizations of the mutual information.

### 6.2. Total Correlation and Dual Total Correlation

Given (S,Y,Z)∼P, the *total correlation (TC)* [115] is defined as follows:

(65) $$\begin{array}{cc} TC(S;Y;Z) & =D(P_{SYZ}∥P_{S}\times P_{Y}\times P_{Z})\\ \; & =H(S)+H(Y)+H(Z)-H(SYZ)\\ \; & =I(S;Y)+I(Y;Z)+I(Z;S)-CoI(S;Y;Z). \end{array}$$

TC measures the total amount of correlations between *S*, *Y*, and *Z*. TC is symmetric in its arguments, non-negative, and vanishes if and only if $P_{SYZ}=P_{S}\times P_{Y}\times P_{Z}$. Total correlation is called multi-information in [116,117] and stochastic interaction in [117].

Te Sun [34] defined a related quantity called the *dual total correlation (DTC)* based on the lattice-theoretic duality of Shannon information measures [32]:

(66) $$\begin{array}{cc} DTC(S;Y;Z) & =H(SYZ)-H(S|YZ)+H(Y|SZ)-H(Z|SY)\\ \; & =I(S;Y|Z)+I(Y;Z|S)+I(Z;S|Y)+CoI(S;Y;Z). \end{array}$$

Like the TC, DTC is symmetric in its arguments, non-negative, and vanishes if and only if $P_{SYZ}=P_{S}\times P_{Y}\times P_{Z}$ [34]. From (65) and (66), we have the following relation between TC and DTC:

(67) $$\begin{array}{c} TC(S;Y;Z)+DTC(S;Y;Z)=I(S;YZ)+I(Y;SZ)+I(Z;SY). \end{array}$$

TC and DTC capture different aspects of the correlations between *S*, *Y*, and *Z*. To see this, consider the Rdn and Xor distributions in Example 1: The correlations in the Rdn distribution can be attributed purely to pairwise interactions since *S*, *Y*, and *Z* are identical random variables. On the other hand, correlations in the Xor distribution arise purely due to triplewise interactions, since *S*, *Y*, and *Z* are pairwise independent. For the Rdn, we have TC=2log2>log2=DTC, and for the Xor, we have DTC=2log2>log2=TC. For distributions where *S*, *Y*, and *Z* have binary supports, TC is maximized by the Rdn distribution, while DTC is maximized by the Xor distribution [117].

Te Sun [34] studied higher-dimensional analogs of these quantities and argued that TC is effective in measuring “local” lower-order correlations, whereas DTC is effective in measuring overall higher-order correlations (see, e.g., Example 6.2 in [34]). For many practical distributions of interest, most of the TC resides in the lower-order correlations [118]. Austin [119] studied the different nature of the structures induced by small values of TC and DTC on a metric space of probability measures: if a joint distribution *P* has a small TC, then *P* is close to a product measure, where closeness is in the sense of the Wasserstein distance; on the other hand, if *P* has a small DTC, then it is close to a mixture of product measures.

**Interconvertibility between probability distributions under LOPC.** Of immediate interest to us are the monotonicity properties of TC and DTC in relation to the problem of converting a given probability distribution to another. Concretely, we consider the following setup: Three collaborating parties, Alice, Bob, and Charlie observe i.i.d. copies of random variables *S*, *Y*, and *Z*, resp., distributed according to some known source distribution *P*. The goal is to convert *P* into a target distribution $P^{\prime }$ when the set of free operations is LOPC by Alice, Bob, and Charlie.

Cerf et al. [120] showed that TC and DTC are monotones under LOPC. In particular, in the tripartite case, TC(S;Y;Z), DTC(S;Y;Z), I(S;YZ), I(Y;SZ), and I(Z;SY) are five monotones. From (67), it is shown that these monotones are not all linearly independent. However, none of these monotones can be expressed as a linear combination of the others with only positive coefficients. Hence, for a given source–target pair, these five monotones set independent constraints on the possible interconversions under LOPC.

Table 1 lists the values of the monotones for some distributions in Example 1. We see, for instance, that the conversion Xor → Rdn is not feasible since TC increases from 1 to 2 while going from Xor to Rdn. Likewise, Rdn → Xor is not feasible since DTC increases from 1 to 2 while going from Rdn to Xor.

On the other hand, the following conversions are feasible and can be achieved using simple protocols [120]:

- Xor → Φ: Charlie publicly announces the value of his bit.
- Rdn → Φ: Charlie forgets his bit (e.g., sends *Z* through a channel that completely randomizes it).
- Xor^⊗2^ → Rdn: Alice, Bob, and Charlie observe, resp., the bits $\left(s,s^{\prime }\right)$, $\left(y,y^{\prime }\right)$, and $\left(z,z^{\prime }\right)$, where z=s⊕y and $z^{\prime }=s^{\prime }⊕y^{\prime }$. Alice publicly announces *s* and Bob $y^{\prime }$. Since Charlie knows $\left(z,z^{\prime }\right)$, she computes z⊕s=y and $z^{\prime }⊕y^{\prime }=s^{\prime }$ and publicly announces $w=y⊕s^{\prime }$. Finally, since Alice knows $s^{\prime }$, she computes $s^{\prime }⊕w=y$. Thus, Alice, Bob, and Charlie end up sharing the bit *y*.
- Rdn^⊗2^ → Xor: Alice, Bob, and Charlie observe, resp., the bits $\left(s,s^{\prime }\right)$, $\left(y,y^{\prime }\right)$, and $\left(z,z^{\prime }\right)$. Alice and Bob forget, resp., *s* and $y^{\prime }$, while Charlie computes $z⊕z^{\prime }$ and forgets the values *z* and $z^{\prime }$.

Cerf et al. [120] considered the more general question of a reversible interconversion between an arbitrary $P_{SYZ}$ and the distributions $\Phi_{SY}$, $\Phi_{YZ}$, $\Phi_{ZS}$, Rdn, and Xor, where we write $\Phi_{SY}$ for the secret bit shared between *S* and *Y*, and likewise for $\Phi_{YZ}$ and $\Phi_{ZS}$. More concretely, does there exist yields $\gamma_{1},\ldots ,\gamma_{5}$ such that the following reversible conversion is feasible under LOPC?

(68) $$\begin{array}{c} P_{SYZ}⇌\Phi_{SY}^{⊗\gamma_{1}}⊗\Phi_{YZ}^{⊗\gamma_{2}}⊗\Phi_{ZS}^{⊗\gamma_{3}}⊗XOR^{⊗\gamma_{4}}⊗RDN^{⊗\gamma_{5}}. \end{array}$$

Cerf et al. [120] showed that the five monotones in Table 1 do not forbid, in principle, the following reversible conversions under LOPC:

- If CoI=0, then $P_{SYZ}⇌\Phi_{SY}^{⊗\gamma_{1}}⊗\Phi_{YZ}^{⊗\gamma_{2}}⊗\Phi_{ZS}^{⊗\gamma_{3}}$.
- If CoI>0, then $P_{SYZ}⇌\Phi_{SY}^{⊗\gamma_{1}}⊗\Phi_{YZ}^{⊗\gamma_{2}}⊗\Phi_{ZS}^{⊗\gamma_{3}}⊗RDN^{⊗\gamma_{5}}$.
- If CoI<0, then $P_{SYZ}⇌\Phi_{SY}^{⊗\gamma_{1}}⊗\Phi_{YZ}^{⊗\gamma_{2}}⊗\Phi_{ZS}^{⊗\gamma_{3}}⊗XOR^{⊗\gamma_{4}}$,

where CoI=SI−CI is the coinformation (see (8)). It is, however, plausible that additional monotones exist that might render some of these conversions infeasible (see, e.g., [84,121]). One natural candidate for such a monotone is an “extractable” version of the function SI in Definition 1, which we describe next.

### 6.3. Extractable Shared Information and Monotonicity Under Local Operations

Rauh et al. [52] and Bertschinger et al. [60] argue that shared information should never increase under local operations (e.g., coarse graining) of the target and/or the predictors. Specifically, for local operations of the predictors, the function SI in Definition 1 satisfies the following property called *right monotonicity* (see A.7 in Appendix A):

(69) $$\begin{array}{c} SI\left(S;Y,Z\right)\geq SI(S;f_{1}\left(Y\right),f_{2}\left(Z\right)) \end{array}$$

for all functions $f_{1}$ and $f_{2}$. However, for local operations on the target, SI does not exhibit a corresponding property, referred to as *left monotonicity* (see A.8 in Appendix A). Rauh et al. [19] proposed a construction that enforces left monotonicity. Define

(70) $$\bar{SI}\left(S;Y,Z\right)=\underset{f:S\to S^{\prime }}{\sup}\;SI\left(f\left(S\right);Y,Z\right),$$

where the supremum runs over all functions $f:S\to S^{\prime }$ from the domain of *S* to an arbitrary finite set $S^{\prime }$. By construction, $\bar{SI}$ satisfies left monotonicity, and $\bar{SI}$ is the smallest function bounded from below by SI that satisfies left monotonicity. One can interpret $\bar{SI}$ as a measure of “extractable” shared information [19]. The intuition is that $\bar{SI}$ is the maximal possible amount of SI one can extract from (Y,Z) by transforming *S* locally. Furthermore, one can generalize the construction to define a probabilistic version of extractability by replacing *f* by a stochastic matrix. This leads to the definition

(71) $$\overset{﹍}{SI}(S;Y,Z):=\underset{P_{S^{\prime }|S}:YZ-S-S^{\prime }}{\sup}SI(S^{\prime };Y,Z).$$

By definition, $\overset{﹍}{SI}$ is monotone under local operations. A study of the monotonicity properties of $\overset{﹍}{SI}$ with respect to public communication is reserved for future study.

**Remark** **7.** *More generally, one can apply the “extractable” construction to arbitrary information measures. Furthermore, by iterating the construction, one can construct an information measure that is monotonic in all arguments *[19]*. An example of this construction is the intrinsic information $I_{↓}$ in *(47)*. The use of *min* instead of *max* in Definition *(47)* reflects that $I_{↓}$ can only increase under local operations by Eve, a monotonicity property it shares with the function UI (see Property P.8 and Proposition 11). Work in a similar vein include *[103]*, where a construction called “arrowing” is used for building probabilistically extractable versions of a given function (see also* [122]*). Galla and Gühne *[123]* discuss probabilistic extractability for a measure of correlation called the “connected correlation” *[124,125,126,127,128]*, which are based on projections onto exponential families *[129]*.*

### 6.4. Left Monotonic Information Decompositions

Is it possible to construct an information decomposition where all measures satisfy left monotonicity? The seemingly simple strategy of starting with an arbitrary decomposition and replacing each partial information measure with its extractable counterpart fails, as this would increase all partial measures (unless already extractable), leading to an overall increase in their sum. For instance, if $\tilde{SI}$ is replaced by a larger function, then $\tilde{UI}$ must be reduced due to constraint (4).

As argued in [52], it is intuitive that $\tilde{UI}$ be left monotonic. In particular, the function UI in Definition 1 satisfies left monotonicity (see Property P.2 in Section 5.4). Likewise, it is also desirable that $\tilde{SI}$ be left monotonic [52,60]. The intuition for synergy is much less clear. The extractable construction cannot be directly generalized to ensure left monotonicity for both unique and shared information. However, such a decomposition may still exist, with left monotonicity affecting the measure of shared information. Suppose that $\tilde{SI}$, $\tilde{UI}$, and $\tilde{CI}$ define a bivariate information decomposition satisfying (4)–(6), and suppose that $\tilde{SI}$ and $\tilde{UI}$ satisfy left monotonicity. Then,

(72) $$\tilde{SI}\left(f\left(Y,Z\right);Y,Z\right)\leq I\left(Y;Z\right)$$

for any function *f* [19]. Inequality (72) is related to the identity axiom (see A.6 in Appendix A). Indeed, it is easy to derive (72) from the identity axiom and from the assumption that $\tilde{SI}$ is left monotonic. None of the non-negative information decompositions proposed so far satisfies (72). Griffith et al. [130] proposed a function $I_{⋏}$ as a measure of shared information that satisfies left monotonicity. However, this function does not induce a non-negative information decomposition (see A.4 in Appendix A).

The next proposition shows that left monotonicity of the shared information is not consistent with the Blackwell property of the unique information:

**Proposition** **14** ([19,20])**.** *There is no bivariate information decomposition satisfying *(4)–(6)* in which $\tilde{UI}$ satisfies the Blackwell property and $\tilde{SI}$ satisfies left monotonicity.*

A resource-theoretic characterization of the complementary information appears challenging. The problem resides with the fact that it is difficult to postulate how the complementary information should behave if, say, one or more parties perform some local operations on their subsystems. Two studies in this direction deserve notice: Rauh et al.’s Section IV.C in [52] show that the measure CI in Definition 1 can either increase or decrease under local operations of the targets and/or the sources. Another work is a decomposition of the total correlation (TC) due to Amari [124]. The total correlation among three variables can be decomposed into a sum of two non-negative terms that quantify, resp., the amount of correlations arising from purely pairwise and purely triplewise interactions [124] (Equation (78)). The latter term can be interpreted as the synergistic component of the total correlation. However, examples are known where this component violates monotonicity under local operations [123]. Finally, an axiomatic approach to information flow in computational systems is motivated in [131], where it is shown that the CI in Definition 1 provides an intuitive and insightful measure of information flow volume. This warrants further investigation.

## Figures and Tables

**Table 1:** Values of the five tripartite monotones for the secret bit Φ, and the Rdn and Xor distributions [120].

	I(S;YZ)	I(Y;SZ)	I(Z;YS)	DTC(S;Y;Z)	TC(S;Y;Z)
Φ	1	1	0	1	1
Rdn	1	1	1	**1**	**2**
Xor	1	1	1	**2**	**1**

## Data Availability

The original contributions presented in the study are included in the article, further inquiries can be directed to the author.

## Appendix A. The Axiomatic Approach to Shared Information

An axiomatic approach to the concept of shared information was pioneered by Williams and Beer [46]. Recall that the shared information can be interpreted as mutual information without the unique information; see (4).

For the general case of *k* finite predictor variables $Y_{1},\ldots ,Y_{k}$, Williams and Beer [46] proposed the *partial information lattice* framework to decompose the total mutual information $I(S;Y_{1},\ldots ,Y_{k})$ into a sum of terms (called *partial information terms*) corresponding to the different ways in which combinations of the variables $Y_{1},\ldots ,Y_{k}$ convey shared, unique, or complementary information about the target *S*. When k=2, writing $Y_{1}\equiv Y$ and $Y_{2}\equiv Z$, the decomposition has the form given by (4)–(6).

The partial information lattice is a consequence of certain natural properties of the shared information (sometimes called the *Williams–Beer axioms*). The underlying idea is that any information about *S* can be classified according to “who knows what”, i.e., which information about *S* is shared by which subsets of $\left\{Y_{1},\ldots ,Y_{k}\right\}$. This idea resonates with *secret-sharing schemes*, a fundamental tool used in many cryptographic protocols [132]. A secret-sharing scheme involves a secret (*S*), a finite set K={1,…,k} of parties, and a family A of (nonempty) subsets of K called the *access structure* that is closed to taking supersets. The goal is to distribute the secret (*S*) among *k* parties such that only elements of A can reconstruct the secret, while all other subsets of K obtain no information about the secret. There is a one-to-one correspondence between the partial information terms of Williams and Beer’s decomposition scheme and the set of access structures of secret-sharing schemes with *k* parties [59].

Let $\tilde{SI}\left(S;Y_{1},\ldots ,Y_{k}\right)$ denote the information about *S* that is shared among the random variables $Y_{1},\ldots ,Y_{k}$. It is natural to demand that $\tilde{SI}$ satisfies the following properties [46]:

A.1 (*Symmetry*). $\tilde{SI}\left(S;Y_{1},\ldots ,Y_{k}\right)$ is symmetric under permutations of $Y_{1},\ldots ,Y_{k}$.
A.2 (*Self-redundancy*). $\tilde{SI}\left(S;Y_{1}\right)=I\left(S;Y_{1}\right)$.
A.3 (*Monotonicity*). $\tilde{SI}\left(S;Y_{1},\ldots ,Y_{k-1},Y_{k}\right)\leq \tilde{SI}\left(S;Y_{1},\ldots ,Y_{k-1}\right)$, with equality if
  $Y_{i}=f\left(Y_{k}\right)$ for some i<k and some function *f*.

We refer to properties A.1–A.3 as the *Williams–Beer axioms*. Any function satisfying these axioms is non-negative [46]. The axioms, however, do not uniquely characterize the function $\tilde{SI}$. When $\tilde{SI}$ is defined, we can associate with each element of the partial information lattice a “local” quantity (the partial information term) that is uniquely determined from $\tilde{SI}$ by a Möbius inversion. The total mutual information $I(S;Y_{1},\ldots ,Y_{k})$ is then a sum of these local terms [46]. In general, however, the local terms can be negative. For a non-negative decomposition, we require the following additional property:

A.4 (*Local positivity*). All the partial information terms in the induced decomposition are non-negative.

Williams and Beer defined a function

(A1) $$\begin{array}{cc} I_{\min}\left(S;Y_{1},\ldots ,Y_{k}\right) & =\sum_{s}P_{S}\left(s\right)\;\min_{i}\left\{\sum_{y_{i}}P_{Y_{i}|S}\left(y_{i}|s\right)\log\frac{P_{S|Y_{i}}\left(s|y_{i}\right)}{P_{S}\left(s\right)}\right\}\\ \; & =\sum_{s}\;\min_{i}\left\{\sum_{y_{i}}P_{SY_{i}}\left(s,y_{i}\right)\log\frac{P_{SY_{i}}\left(s,y_{i}\right)}{P_{S}\left(s\right)P_{Y_{i}}\left(y_{i}\right)}\right\}, \end{array}$$

and showed that $I_{\min}$ satisfies A.1–A.3 and that the decomposition induced by $I_{min}$ satisfies A.4. While the measure $I_{\min}$ has subsequently been criticized for “not measuring the right thing” [26,53,60], there has been no successful attempt to find better measures, except for the bivariate case (k=2) [6,26]. One problem seems to be the lack of a clear consensus on the values of the shared information for some paradigmatic examples. For example, it seems natural that the shared information is zero for the Copy distribution in Example 1 since *Y* and *Z* are independent [6,26,60]. However, $I_{\min}$ assigns 1 bit of shared information in this case. The second problem relates to the difficulty of coming up with a minimal number of “natural” and “essential” properties for the shared information.

Bertschinger et al. [60] proposed the following additional axiom:

A.5 (*Left chain rule*). $\tilde{SI}\left(SS^{\prime };Y_{1},\ldots ,Y_{k}\right)=\tilde{SI}\left(S;Y_{1},\ldots ,Y_{k}\right)+\tilde{SI}\left(S^{\prime };Y_{1},\ldots ,Y_{k}|S\right)$, where $\tilde{SI}\left(S^{\prime };Y_{1},\ldots ,Y_{k}|S\right)=\sum_{s\in S}P_{S}\left(s\right)\tilde{SI}\left(S^{\prime };Y_{1},\ldots ,Y_{k}|s\right)$.

A.5 is a natural generalization of the chain rule of mutual information (3).

Specializing to the bivariate case, A.4 and A.5 together imply the following property [60], which was first proposed in [26]:

A.6 (*Identity*). $\tilde{SI}\left(\left(Y,Z\right);Y,Z\right)=I\left(Y;Z\right)$.

The identity property states that if *S* is an identical copy of the predictor variables, i.e., if S=(Y,Z), then the shared information should equal the mutual information between *Y* and *Z*. Rauh et al. [52], however, showed that A.6 is incompatible with A.4 for k≥3. This implies that A.4 and A.5 are not compatible for k≥3.

Rauh et al. [52] argue that shared information should never increase if the target and/or the predictors perform some *local* operation (e.g., coarse graining) on their subsystems. For local operations of the predictors, the Williams–Beer axioms imply the following property:

A.7 *(Right monotonicity). $\tilde{SI}\left(S;Y_{1},\ldots ,Y_{k}\right)\geq \tilde{SI}\left(S;f_{1}\left(Y_{1}\right),\ldots ,f_{k}\left(Y_{k}\right)\right)$ for all functions $f_{1},\ldots ,f_{k}$.*

On the other hand, the left chain rule A.5 implies the following property [60]:

A.8 (*Left monotonicity*). $\tilde{SI}\left(S;Y_{1},\ldots ,Y_{k}\right)\geq \tilde{SI}\left(f\left(S\right);Y_{1},\ldots ,Y_{k}\right)$ for all functions *f*.

## Appendix B. Deferred Proofs

**Proof of Lemma** **4.** Since $Z{⊒}_{S}Y$, there exists some $\lambda^{\prime }\in M\left(Z;Y\right)$ such that $\kappa =\lambda^{\prime }\circ \mu$. Hence,

$$\begin{array}{cc} \delta_{o}^{\pi }\left(\kappa ,\nu \right) & =\min_{\lambda \in M(Y;W)}D(\nu ∥\lambda \circ \kappa |\pi_{S})\\ \; & =\min_{\lambda \in M(Y;W)}D(\nu ∥\lambda \circ \lambda^{\prime }\circ \mu \left|\pi_{S}\right)\\ \; & \geq \min_{\lambda \in M(Z;W)}D(\nu ∥\lambda \circ \mu |\pi_{S})=\delta_{o}^{\pi }\left(\mu ,\nu \right). \end{array}$$

Since $Y{⊒}_{S}W$, there exists some $\lambda^{\prime }\in M\left(Y;W\right)$ such that $\nu =\lambda^{\prime }\circ \kappa$. Hence,

$$\begin{array}{cc} \delta_{o}^{\pi }\left(\mu ,\nu \right) & =\min_{\lambda \in M(Z;W)}D(\nu ∥\lambda \circ \mu |\pi_{S})\\ \; & =\min_{\lambda \in M(Z;W)}D(\lambda^{\prime }\circ \kappa ∥\lambda \circ \mu |\pi_{S})\\ \; & \leq \min_{\lambda \in M(Z;Y)}D(\lambda^{\prime }\circ \kappa ∥\lambda^{\prime }\circ \lambda \circ \mu \left|\pi_{S}\right)\\ \; & \leq \min_{\lambda \in M(Z;Y)}D(\kappa ∥\lambda \circ \mu |\pi_{S})=\delta_{o}^{\pi }\left(\mu ,\kappa \right), \end{array}$$

where the inequality in the last step follows from the data processing inequality for the KL divergence (Theorem 2.2 in [28]). □

**Proof of Proposition** **11.** We shall use the following variational characterization of the UI, which follows from Property P.8:

(A2) $$\begin{array}{c} UI\left(S;Y\backslash Z\right)=\min_{P_{Z^{\prime }|Z}:SY-Z-Z^{\prime }}UI\left(S;Y\backslash Z^{\prime }\right). \end{array}$$

Let (S,Y,Z)∼P and let $\left(S,Y,Z^{\prime }\right)∼P^{\prime }$ such that $P^{\prime }=P\cdot P_{Z^{\prime }|Z}\equiv \sum_{z\in Z}P_{SYZ}P_{Z^{\prime }|Z}$. Let $Q\in \Delta_{P}$ and $Q^{\prime }=Q\cdot P_{Z^{\prime }|Z}\in \Delta_{P^{\prime }}$. We then have

$$\begin{array}{cc} UI(S;Y\backslash Z) & =\min_{P_{Z^{\prime }|Z}:SY-Z-Z^{\prime }}UI\left(S;Y\backslash Z^{\prime }\right)\\ \; & =\min_{P_{Z^{\prime }|Z}:SY-Z-Z^{\prime }}\min_{Q^{\prime }\in \Delta_{P^{\prime }}}I_{Q^{\prime }}\left(S;Y|Z^{\prime }\right)\\ \; & \leq \min_{P_{Z^{\prime }|Z}:SY-Z-Z^{\prime }}\min_{Q\in \Delta_{P}}I_{Q\cdot P_{Z^{\prime }|Z}}\left(S;Y|Z^{\prime }\right)\\ \; & =\min_{Q\in \Delta_{P}}\min_{P_{Z^{\prime }|Z}:SY-Z-Z^{\prime }}I_{Q\cdot P_{Z^{\prime }|Z}}\left(S;Y|Z^{\prime }\right)\\ \; & =\min_{Q\in \Delta_{P}}I_{Q}\left(S;Y\;\;↓\;Z\right)\leq I_{P}\left(S;Y\;\;↓\;Z\right), \end{array}$$

where the first step is just (A2), and the inequality in the third step follows since for any $Q\in \Delta_{P}$, $Q^{\prime }$ lies in $\Delta_{P^{\prime }}$. □
